# 4-Aminoquinoline derivatives and benzoxaborole-4-aminoquinoline hybrids: synthesis, antiplasmodial evaluation, heme-inhibition and *in silico* studies

**DOI:** 10.1039/d6ra02824a

**Published:** 2026-07-08

**Authors:** Anu Saini, Sumit Kumar, Bruno Pradines, Océane Delandre, Joel Mosnier, Isabelle Fonta, Yukta Soni, Patrick Appiah-Kubi, Ashona Singh, Raghu Raj, Vipan Kumar

**Affiliations:** a Department of Chemistry, Guru Nanak Dev University Amritsar India vipan_org@yahoo.com; b Department of Biomedicine, University of Bergen 5007 Bergen Norway; c Department of Chemistry, University of Bergen Allégaten 41 5007 Bergen Norway; d Unité Parasitologie et Entomologie, Departement Risques Infectieux, Institut de Recherche Biomédicale des Armées Marseille France; e Aix Marseille Univ, SSA, AP-HM, RITMES Marseille France; f IHU Méditerranée Infection Marseille France; g Centre National de Référence du Paludisme Marseille France; h PG Department of Chemistry, DAV College Amritsar India raghusharma4@gmail.com; i Department of Chemistry, University of Pretoria Gauteng South Africa

## Abstract

A library of 4-aminoquinoline derivatives and benzoxaborole-4-aminoquinoline hybrids, linked through amide and 1*H*-1,2,3-triazole spacers, was synthesized and evaluated against both chloroquine-sensitive (3D7) and -resistant (W2) strains of *Plasmodium falciparum*. Structure–activity relationship studies revealed that antiplasmodial potency was strongly influenced by the length of the alkyl chain and by the nature of the terminal functional group (azido, aldehyde, or benzyl alcohol). Notably, incorporation of the benzoxaborole core significantly enhanced activity relative to the parent 4-aminoquinoline derivatives. Among the series, hybrid 8b emerged as the most potent analogue, displaying superior activity against the CQ-resistant W2 strain compared with the reference antimalarials quinine and chloroquine. The hybrids exhibited negligible cytotoxicity toward HEK-293 cells, affording selectivity indices of up to ∼700. UV-visible spectroscopic titrations demonstrated that compound 8b binds monomeric heme more selectively than CQ at both physiological and digestive vacuole pH, supporting inhibition of hemozoin formation as its primary mode of action. Furthermore, homology modelling, induced-fit docking, and molecular dynamics simulations with both wild-type and benzoxaborole-resistant (H36Y/D470N) PfCPSF3 suggested that the scaffold can maintain a stable Zn^2+^-coordinated binding mode in both protein variants. These findings indicate a potential dual mechanism involving hemozoin inhibition and PfCPSF3 targeting, warranting further experimental validation.

## Abstract 2

A library of 4-aminoquinoline derivatives and benzoxaborole-4-aminoquinoline hybrids was synthesized and evaluated against chloroquine-sensitive (3D7) and -resistant (W2) strains of *Plasmodium falciparum*.

## Graphical abstract

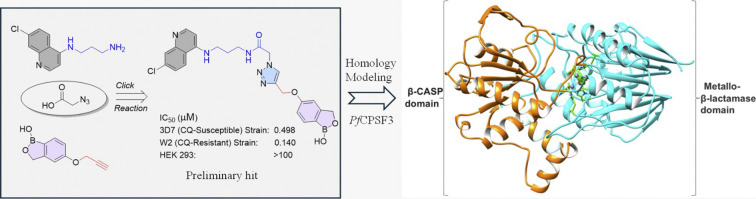

## 1 Introduction

Malaria, one of the oldest hematologic diseases, is caused by protozoan parasites of the genus *Plasmodium* and transmitted through the bite of infected a female Anopheles mosquito. Despite the substantial advancements in medicine and public health, malaria continues to pose a major global health challenge, particularly in tropical and subtropical regions where environmental conditions support vector proliferation. According to recent World Health Organization reports, approximately 282 million malaria cases and an estimated 610 000 malaria-related deaths were recorded worldwide in 2024. Sub-Saharan Africa noted the greatest burden, especially among children under the age of five.^1^ A complex interplay of geographic, climatic, and socioeconomic factors drive the persistence and severity of malaria. The disease remains highly endemic across Africa, Southeast Asia, parts of the Americas and the Western Pacific. Among the *Plasmodium* species, *Plasmodium falciparum* is the most lethal, responsible for severe complications such as cerebral malaria and multi-organ failures. It accounts for most of the malaria-related mortality.^2,3^

Although several classes of antimalarial drugs, which include antibiotics, antifolates, endoperoxides, aminoquinoline and non-aminoquinoline analogues, are widely used, the emergence and rapid spread of drug-resistant *P. falciparum* have impeded global malaria control and eradication efforts.^4,5^ The escalating resistance highlights the urgent need for innovative therapeutic strategies, including the development of new chemical entities and hybrid molecules to effectively combat this life-threatening disease. Among the available antimalarials, 4-aminoquinolines have long served as a cornerstone scaffold in malaria treatment.^6^ In the early twentieth century, CQ was developed as the prototypical member of this class due to its remarkable efficacy, low toxicity, and affordability. CQ exerts its antimalarial effects by inhibiting haemozoin formation within the parasite's digestive vacuole, thereby disrupting the detoxification of heme generated during haemoglobin degradation.^7^

Despite their historical success, the clinical utility of 4-aminoquinolines has been severely compromised by the widespread emergence of chloroquine-resistant *P. falciparum*.^8^ Importantly, research indicates that resistance is often compound-specific, suggesting that strategic structural modifications within the 4-aminoquinoline framework may restore or even enhance activity against resistant parasites.^9–11^ Consequently, the design and synthesis of new analogues and hybrid molecules based on this privileged scaffold remain central to contemporary antimalarial drug discovery, underscoring its continued relevance in the search for next-generation therapeutics.^12^

Benzoxaboroles, cyclic hemi-esters of boronic acids, have emerged as a highly versatile scaffold that has attracted considerable attention within the scientific community, particularly following the FDA approval of the antifungal tavaborole.^13^ These molecules exhibit exceptional physicochemical and drug-like properties, including low toxicity and high target specificity.^14^ Benzoxaboroles have been extensively explored for the design of antimalarial,^15^ antibacterial,^16^ antitrypanosomal,^17^ antitubercular,^18^ and anticancer agents.^19^ Notably, both classes of boronic acids and benzoxaboroles decompose into boric acid, which highlight their attractive low biotoxicity in drug design. Literature reports emphasize that the presence of a boron atom in this moiety is essential for antimalarial potential. Therefore, these compounds are considered green or environmentally friendly molecules.^20^

In our recent work, we investigated the synthesis and antiplasmodial efficacy of triazole-linked benzoxaborole analogues and 4-aminoquinoline-benzoxaborole hybrids against both chloroquine-susceptible and -resistant strains of *P. falciparum*. Structure–activity relationship (SAR) analysis revealed that the incorporation of a quinoline moiety significantly enhanced antiplasmodial activity, and that flexible alkyl chains were preferred as linkers over rigid piperazine units. Furthermore, the 4-aminoquinoline-benzoxaborole hybrids exhibited superior potency compared to the benzoxaborole analogues, particularly against the W2-resistant strain.^21^ In continuation of our efforts to identify effective antiplasmodials, the present manuscript describes the design and synthesis of a new series of 4-aminoquinoline-benzoxaborole hybrids linked *via* either an amide or 1*H*-1,2,3-triazole linker, along with their biological evaluation against the chloroquine-sensitive 3D7 and chloroquine-resistant W2 strains of *P. falciparum*.

## 2 Results and discussion

### 2.1 Synthetic chemistry

**Scheme 1:**
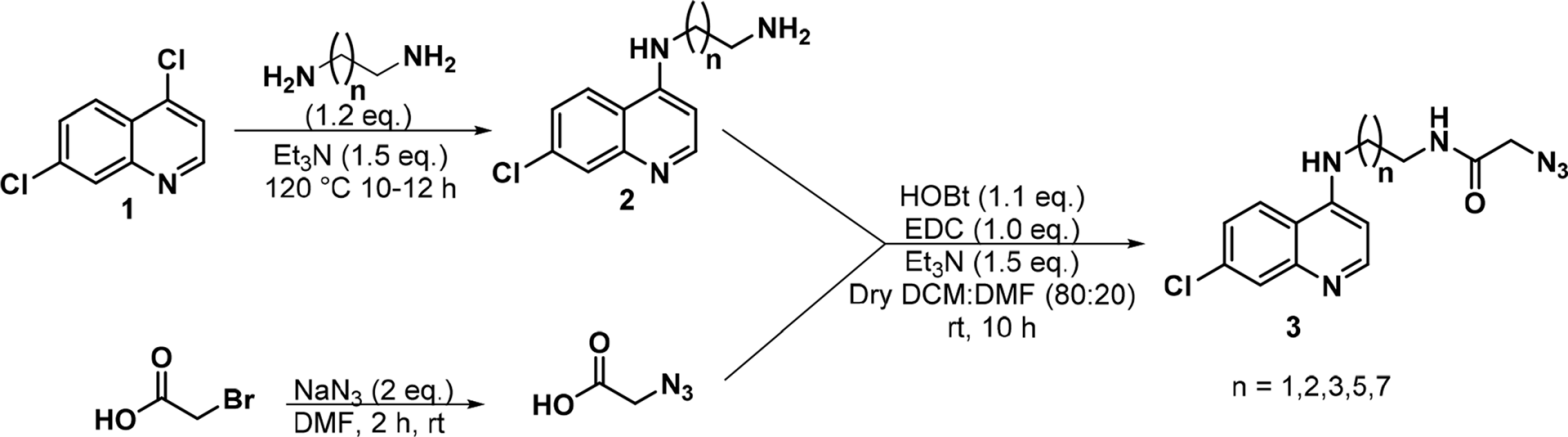
Synthesis of amide-linked azidoquinoline derivatives 3.

4-Aminoquinolines 2 were synthesized from 4,7-dichloroquinoline 1 by reacting it with an excess of the corresponding diamines at 120 °C for 7–8 hours. Distinctly, the reaction of bromoacetic acid with sodium azide at room temperature for 2 hours afforded azidoacetic acid. The synthesized 4-aminoquinolines 2 were subsequently subjected to amide coupling with azidoacetic acid using HOBt-EDC as coupling reagents, resulting in the formation of the amide-tethered azidoquinoline derivatives 3 (Scheme 1).

**Scheme 2:**
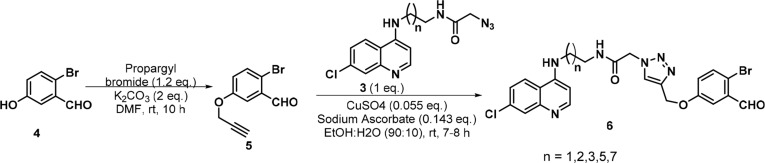
Preparation of amide-triazole-linked precursors 6.

2-Bromo-3-hydroxybenzaldehyde 4, prepared from 3-hydroxybenzaldehyde *via* bromination in dry DCM, was subsequently subjected to propargylation under K_2_CO_3_/DMF conditions at room temperature to afford the second precursor, *O*-propargylated bromobenzaldehyde 5. The synthesized precursors, amide-tethered azidoquinolines 3, were then subjected to a CuSO_4_/sodium ascorbate-promoted azide–alkyne cycloaddition with *O*-propargylated bromobenzaldehyde 5, yielding the triazole-linked intermediate compounds 6, as depicted in Scheme 2.

**Scheme 3:**
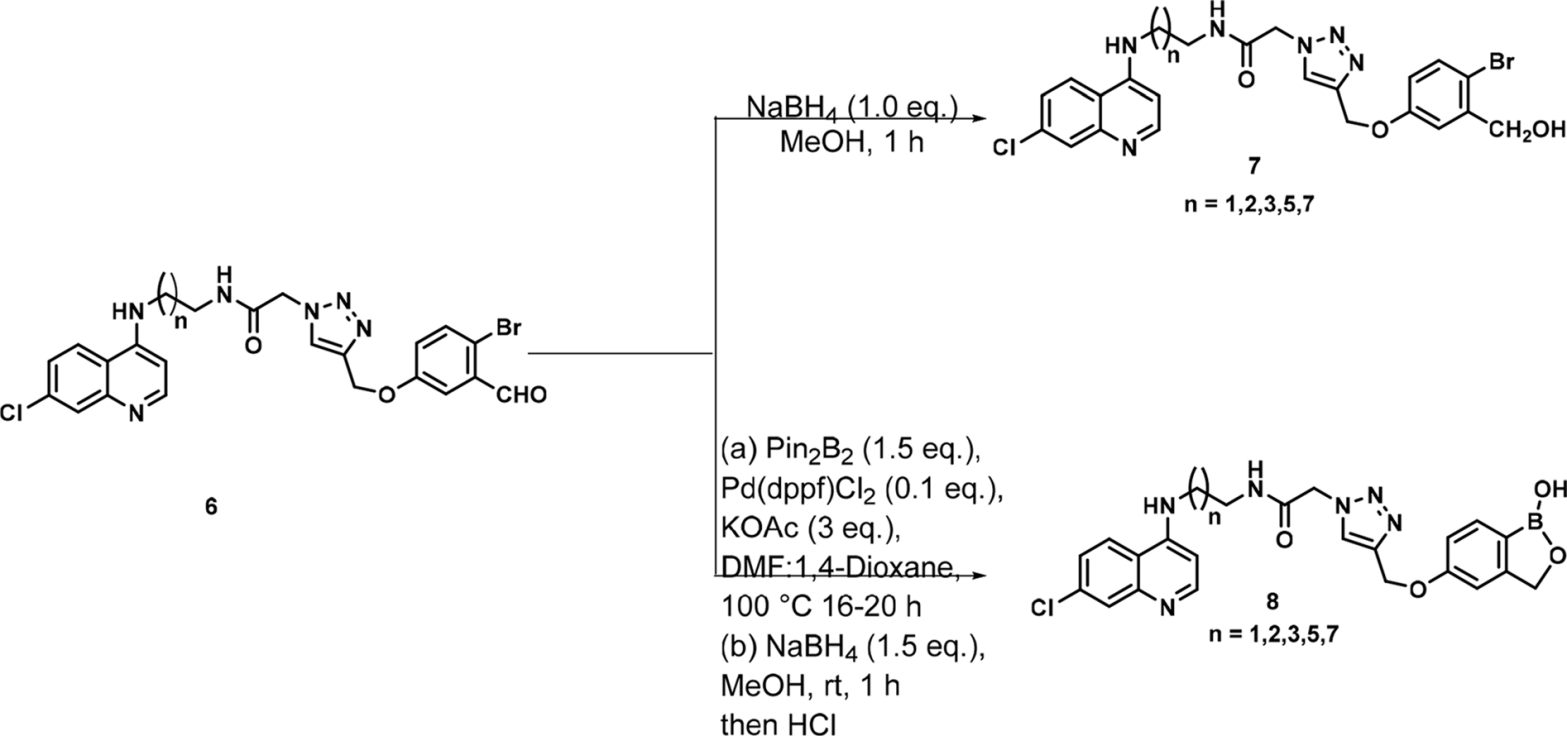
Synthesis of 7-chloroquinoline-based alcohol derivatives 7 and 7-chloroquinoline-benzoxaborole hybrids 8.

Further, palladium-catalyzed borylation of 6 using bis(pinacolato)diboron and potassium acetate as the base in 1,4-dioxane at 100 °C afforded the amide- and triazole-linked 7-chloroquinoline-based boronic esters. Subsequent reduction with sodium borohydride in dry methanol at room temperature for 1 hour, followed by acidification, yielded the corresponding amide-triazole-tethered chloroquinoline-benzoxaborole hybrids 8. To enable comparison of the biological activity between the pure organic ligands and their benzoxaborole hybrids, compound 6 was also reduced with sodium borohydride in dry methanol to furnish the amide-triazole-linked chloroquinoline-based benzyl alcohol derivatives 7 (Scheme 3).

The structures of synthesized compounds were assigned based on spectral data and analytical evidence. For example, the benzoxaborole-4-aminoquinoline compound 8d showed molecular ion peaks [M + 1] and [M + 3] at 549.2118 and 549.2120 in its HRMS spectrum. In the ^1^H NMR spectrum, the compound exhibited singlet peaks at *δ* 4.45, 5.10 and 5.15 due to the presence of three methylene groups, multiplets at *δ* 1.32–1.46, 1.66–1.69, 3.09–3.12, and 3.48–3.52 due to the presence of six methylene groups of alkyl chain, and singlet at *δ* 8.17 corresponding to the characteristic triazole proton. Further, the ^13^C NMR spectrum revealed a characteristic signal at *δ* 165.6 due to carbonyl carbon along with the requisite number of carbons confirming the assigned structure.

### 2.2 *In vitro* antiplasmodial activity

Twenty compounds were evaluated for their antiplasmodial activities against the CQ-susceptible 3D7 and CQ-resistant W2 strains of *P. falciparum*. The 50% inhibitory concentrations (IC_50_) were estimated through non-linear regression of the results of one replicate of 11 drug concentrations. The antiplasmodial *in vitro* assay was performed five times for each product. The IC_50_ values represented the mean value (± standard deviation) calculated from the five independent experiments. The biological data is summarized in Table 1 and benchmarked against a panel of reference antimalarial drugs. A systematic structure–activity relationship (SAR) analysis demonstrated that the antiplasmodial potency strongly depends on both the alkyl chain length and the core structure of the synthesized adducts. Against the CQ-susceptible 3D7 strain, the quinoline-based azides 3a–e displayed modest activity, with IC_50_ values ranging from 2.16 to 5.10 µM. A general increase in activity was observed as the chain length increased from C-2 to C-6, except for 3b. However, extending the chain from C-6 to C-8 led to a decline in potency, which may be attributed to excessive lipophilicity adversely affecting biological interaction.

**Table 1:** Antiplasmodial activities of synthesized compounds on CQ-susceptible 3D7 and CQ-resistant W2 strains of *P. falciparum*

Compound	Spacer (*n*)	IC_50_ (µm) 3D7 strain^a^	IC_50_ (µm) W2 strain^b^	RI^c^
3a	2	5.104 ± 0.091	5.417 ± 0.352	1.06
3b	3	2.199 ± 0.101	4.337 ± 0.253	1.97
3c	4	4.587 ± 0.246	4.702 ± 0.192	1.02
3d	6	2.161 ± 0.225	4.394 ± 0.192	2.03
3e	8	4.638 ± 0.943	7.158 ± 0.429	1.54
6a	2	3.862 ± 0.569	3.966 ± 0.610	1.02
6b	3	1.672 ± 0.081	1.406 ± 0.450	0.84
6c	4	0.825 ± 0.134	0.842 ± 0.076	0.09
6d	6	0.422 ± 0.053	0.217 ± 0.022	0.51
6e	8	0.438 ± 0.099	0.488 ± 0.116	1.11
7a	2	3.980 ± 0.344	1.527 ± 0.098	0.38
7b	3	3.430 ± 0.537	2.837 ± 0.389	0.83
7c	4	2.791 ± 0.323	4.037 ± 0.080	1.44
7d	6	0.578 ± 0.069	0.159 ± 0.042	0.27
7e	8	4.355 ± 0.278	4.890 ± 0.119	1.12
8a	2	0.330 ± 0.054	0.578 ± 0.118	1.75
8b	3	0.498 ± 0.036	0.140 ± 0.019	0.28
8c	4	0.318 ± 0.076	0.290 ± 0.030	0.91
8d	6	0.185 ± 0.029	0.176 ± 0.042	0.95
8e	8	0.208 ± 0.054	0.175 ± 0.039	0.84
Chloroquine	—	0.019 ± 0.005	0.475 ± 0.137	23.8
Desethylamodiaquine	—	0.016 ± 0.004	0.121 ± 0.021	7.45
Mefloquine	—	0.062 ± 0.10	0.024 ± 0.005	0.38
Quinine	—	0.161 ± 0.028	0.504 ± 0.058	3.12
Dihydroartemisinin	—	0.002	0.001	0.82

a CQ-S: Chloroquine-susceptible strain.

b CQ-R: Chloroquine-resistant strain.

c Resistance index (RI): IC_50_(W2)/IC_50_(3D7) according to chloroquine susceptibility. The IC_50_ values were the mean of five independent experiments (± standard deviation).

Conversion of these azides to the corresponding 1*H*-1,2,3-triazole-linked bromobenzaldehydes (6a–e) resulted in a marked enhancement in activity, with IC_50_ values ranging from 0.422–3.862 µM. Among these, the aldehydes bearing butyl (6c), hexyl (6d), and octyl (6e) linkers were the most potent, exhibiting IC_50_ values of 0.825 ± 0.134, 0.422 ± 0.053, and 0.438 ± 0.099 µM, respectively. Subsequent reduction of these arylaldehydes afforded the corresponding alcohols 7a–e, which showed activities comparable to or slightly lower than their aldehyde counterparts. Within this series, 7d (hexyl linker) emerged as the most active (IC_50_ = 0.578 ± 0.069 µM), while 7e deviated from the trend and showed a pronounced loss of activity. The benzoxaborole hybrids 8a–e demonstrated significant improvement in potency relative to their precursors, with IC_50_ values ranging from 0.185 to 0.498 µM. Notably, compounds 8d and 8e, incorporating hexyl and octyl linkers, exhibited the highest activities with IC_50_ values of 0.185 ± 0.029 and 0.208 ± 0.064 µM, respectively. Although these compounds were approximately ten-fold less potent than chloroquine against the sensitive strain, they were found to be equipotent to quinine.

A similar trend in activity was observed against the chloroquine-resistant W2 strain, where antiplasmodial potency correlated with the alkyl chain length. For compounds 3a–e and 6a–e, antiplasmodial efficacy increases with increasing length of alkyl chain from C-2 to C-6 and then decreases on going from C-6 to C-8 against this strain, except for 3c. However, in case of compounds 7a–e, potency declines with increasing length of alkyl chain with exception of 7d. There was no correlation found in case of compound 8a–e against W2 strain.

As previously noted, the quinoline-based azides 3a–e were essentially inactive against W2 strain, whereas their corresponding triazole-linked bromo-aldehydes (6a–e) displayed a substantial improvement in activity. Among these, 6d and 6e exhibited notable potency, with IC_50_ values (against W2 Strain) of 0.217 ± 0.022 and 0.488 ± 0.116 µM, respectively. The reduced analogues (7a–e) showed activities that were either slightly lower or comparable to those of the aldehydes. Within this series, 7d (*n* = 6) emerged as the most active, displaying an IC_50_ of 0.159 ± 0.042 µM. The synthesized benzoxaboroles (8a–e) were consistently active on the resistant strain, with potency improving as the alkyl chain length increased. The hybrids 8b (*n* = 2), 8d (*n* = 6), and 8e (*n* = 8) were the most potent members of the series, exhibiting IC_50_ values of 0.140 ± 0.019, 0.176 ± 0.042, and 0.175 ± 0.039 µM, respectively. Notably, the most active compound, 8b, was approximately three-fold more potent than the drug standards chloroquine and quinine and demonstrated activity comparable to that of desethylamodiaquine on the resistant strain.

All synthesized compounds exhibited markedly reduced cross-resistance compared with the standard antimalarial drugs chloroquine, desethylamodiaquine, and quinine, with resistance index (RI) values ranging from 0.09 to 2.12, in contrast to 23.8 for chloroquine, 7.45 for desethylamodiaquine, and 3.12 for quinine. Notably, many of the synthesized compounds retained strong antiplasmodial activity against the chloroquine-resistant strain. Among them, compounds 6c, 7d, and 8b demonstrated outstanding RI values, indicating minimal susceptibility to resistance mechanisms. In particular, the most potent conjugate, 8b, displayed the lowest RI value (0.28), highlighting its superior resistance tolerance and strong potential to overcome chloroquine resistance.

The most promising hybrids 8a–8e were screened for their cytotoxicity against human embryonic kidney HEK-293 cell lines, and the results are shown in Table 2. All compounds 8a–8e were non-cytotoxic against the normal human cell line and exhibited high values of selective indices (SI) in the range of 200–714. The most active compound, 8b, exhibited selectivity of >200 for the CQ-sensitive strain and >714 for the W2-resistant strain.

**Table 2:** Cytotoxicity of compounds 8a–8e against human embryonic kidney cells

Compound	Spacer	IC_50_ (µM) HEK-293 (µM)	IC_50_ (µm) 3D7 strain	IC_50_ (µm) W2 strain	SI of 3D7 strain	SI of W2 strain
8a	2	>100	0.330	0.578	>303	>173
8b	3	>100	0.498	0.140	>200	>714
8c	4	99.54	0.318	0.290	313	343
8d	6	52.70	0.185	0.176	284	299
8e	8	>100	0.208	0.175	480	>571

**Fig. 1:**
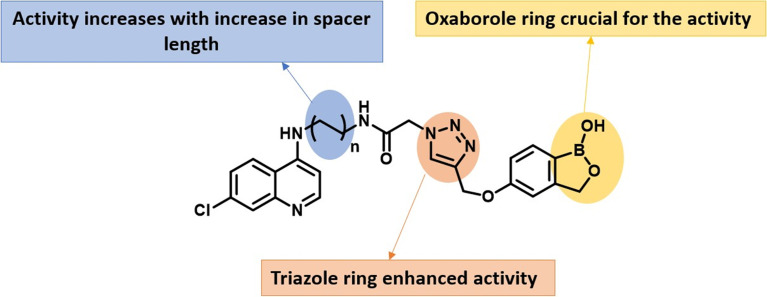
Graphical structure–activity relationship.

The graphical structure activity relationship is depicted in Fig. 1.

### 2.3 Hemin-inhibition studies

**Fig. 2:**
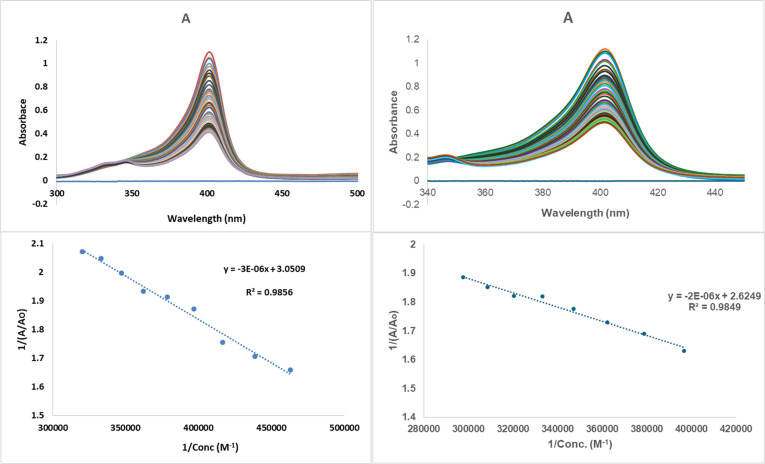
Titration of monomeric heme (12 µM) with increasing concentration of compound 8b (0–38 µM) along with linear dependence of absorption at 401.5 nm on heme-8b complex concentration in aqueous DMSO solution at (A) pH 7.4, 0.02 M HEPES buffer and (B) pH 5.6, 0.02 M MES buffer, respectively.

The mode of action of most antimalarial agents, particularly 4-aminoquinoline-based compounds, involves targeting monomeric heme, generated during the hemoglobin-digestion pathway, within the parasite's food vacuole. Free heme (Fe^3+^ ferriprotoporphyrin IX) is highly toxic to *Plasmodium* and is normally detoxified through polymerization into inert hemozoin crystals. 4-Aminoquinoline derivatives disrupt this detoxification process by forming stable complexes with Fe^3+^ ferriprotoporphyrin IX *via* π–π stacking interactions, thereby inhibiting hemozoin formation and leading to parasite death. Heme complexation by antimalarial compounds in aqueous media can be monitored through characteristic changes in the UV-visible spectrum of aqueous hematin. In view of the promising antiplasmodial activity exhibited by the synthesized compounds, heme-binding studies were performed on the most active benzoxaborole-4-aminoquinoline conjugate, 8b, to gain insight into its possible mechanism of action. To further elucidate its binding behavior, the interaction of compound 8b with hemin chloride was investigated using spectrophotometric titrations conducted at pH 7.4 (physiological conditions) and pH 5.6, which mimics the acidic environment of the parasite's food vacuole. Spectrophotometric titrations were carried out by the stepwise addition of increasing concentrations of compound 8b (0–38 mM, 40% DMSO) to a constant concentration of free heme (12 µM) in 0.02 M HEPES buffer (pH 7.4) and 0.02 M MES buffer (pH 5.6). A progressive decrease in the intensity of the characteristic heme absorption band was observed, confirming the formation of a heme-8b complex, as depicted in Fig. 2. Binding constants were subsequently determined by fitting the titration data using HypSpec, a nonlinear least-squares fitting program. As summarized in Table 3, compound 8b exhibited stronger heme-binding affinity than chloroquine at both physiological and acidic pH, supporting its proposed hemozoin-inhibition-based mechanism and enhanced antiplasmodial efficacy.

**Table 3:** Binding constant (log *K*) for 8b and CQ with monomeric heme

Compound	pH 7.4 (HEPES buffer)	pH 5.6 (MES buffer)
8b	6.00	6.02
CQ	5.18	5.10

### 2.4 *In silico* physicochemical and drug-likeness profiling

The physicochemical and drug-like properties of potent benzoxaborole-4-aminoquinoline conjugates 8a–8e were evaluated *in silico* to access their potential effectiveness and safety. The predictions were performed using the small molecule physicochemical and drug-likeness descriptor module, accessible at https://www.swissadme.ch/ and https://biosig.unimelb.edu.au/pkcsm/prediction.

The evaluated compounds satisfied several key Lipinski parameters, particularly lipophilicity, all analogues displayed log *P* values below 5, suggesting the potential for favorable membrane permeability (Table 4). A gradual increase in molecular weight was observed across the series, with compounds 8b–e exceeding the conventional 500 Da threshold, accompanied by an increase in the number of rotatable bonds beyond 10. Despite these deviations, all compounds showed a constant polar surface area (TPSA) of 123.42 Å^2^, well below the 140 Å^2^ limit associated with acceptable oral absorption, whilst maintaining moderate hydrogen bond donor (HBD = 3) and acceptor (HBA = 7) counts. Consequently, the higher-molecular-weight analogues fall within the “beyond Rule of Five” (bRo5) chemical space, a framework that recognizes the limitations of Lipinski criteria for structurally complex and target-engaging molecules. Within this space, deviations in molecular weight and conformational flexibility are considered acceptable, provided that key parameters such as lipophilicity and TPSA remain within ranges compatible with membrane permeation. The bRo5 characterizes the oral bioavailability of the compounds through favorable intramolecular interactions and conformational adaptability, thereby reducing the effective exposure of polar functionalities during membrane transit.^22^ This concept is supported by clinically successful drugs targeting challenging biological systems, highlighting the drug-likeness and translational potential of the present analogues despite violating Lipinski's rule.

**Table 4:** Physicochemical and lipophilicity of the most active compounds

Compound	Lipinski's rule					TPSA (Å^2^)
	log *P* < 5	MW < 500	HBD < 5	HBA < 10	RB < 10	
8a	1.5047	492.73	3	7	10	123.42
8b	1.8948	506.75	3	7	11	123.42
8c	2.2849	520.78	3	7	12	123.42
8d	3.0651	548.83	3	7	14	123.42
8e	3.8453	576.88	3	7	16	123.42

### 2.5 Homology modelling

**Fig. 3:**
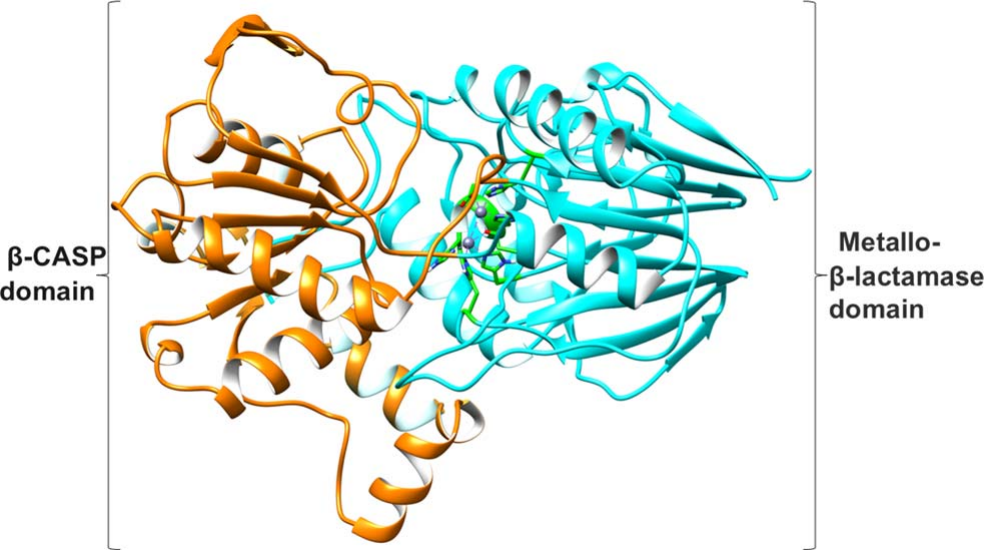
The modelled three-dimensional structure of PfCPSF3 shown in cartoon representation, highlighting the two major catalytic domains: the β-CASP domain (orange) and the metallo-β-lactamase domain (cyan). The catalytic site located at the interface of these domains contains zinc metal ions coordinated by conserved residues (green), shown in stick representation.

*P. falciparum* cleavage and polyadenylation specificity factor subunit 3 (PfCPSF3) is a validated antimalarial target of benzoxaborole-based compounds.^23,24^ Accordingly, PfCPSF3 was selected as the putative target for computational evaluation of the benzoxaborole-4-aminoquinoline hybrids 8a–8e. The three-dimensional (3D) structure of PfCPSF3 was successfully modelled using Schrodinger Maestro homology builder, and evaluated for structural reliability. The predicted model shared ∼51% sequence identity and coverage with the template structure, *Cryptosporidium hominis* CPSF3, is generally considered acceptable for reliable homology modelling.^25^ The level of sequence identity is notably higher than the ∼39% and ∼27% template identities reported in a previous study.^26^ Structural validation using the MolProbity and ProSA server indicated good stereochemical quality.^27^ The modelled structure exhibited a MolProbity Z-score of 1.31, which lies within the acceptable threshold of <2, confirming the structural reliability of the predicted PfCPSF3 model. Ramachandran plot analysis showed that 93.65% of residues were in the favored regions and 99.12% in the allowed regions, with 0.88% classified as outliers (Fig. S1 and Table S2). The modelled PfCPSF3 structure is presented in Fig. 3. To obtain the *Plasmodium falciparum* CPSF3 (PfCPSF3) W2 resistant strain, the double H36Y/D470N mutation was introduced, as it has been shown to present high-level resistance to benzoxaborole antimalarial compounds.^23^

### 2.6 Induced fit molecular docking of wild-type and mutant PfCPSF3

Induced-fit docking (IFD) was conducted to predict the potential binding modes and interaction profiles of 8a–8e with *Plasmodium falciparum* CPSF3. Both the wild-type (WT) and mutant (MT) forms of the protein were evaluated. Chloroquine (cq^+^) and quinine (qn) were included as reference antimalarial compounds. The IFD scoring function accounts for both ligand flexibility and protein structural adaptability, thereby providing a more comprehensive estimate of ligand–protein binding affinity compared with conventional rigid docking approaches.^28,29^ The IFD results are summarized in Table 5. Comparison of the IFD-derived binding affinity scores for compounds 8a–8e against wild-type and mutant PfCPSF3 reveals enhanced predicted binding to the mutant protein (−1021.16 to −1017.15 kcal mol^−1^) relative to the wild-type (−1013.13 to −1007.52 kcal mol^−1^). Notably, 8a–8b outperformed the reference antimalarial drugs chloroquine (cq^+^) and quinine (qn) at PfCPSF3-MT (−1012.33 and −1006.58 kcal mol^−1^, respectively) and PfCPSF3-WT (−1003.22 and −999.42 kcal mol^−1^, respectively). This differential behaviour suggests that the H36Y/D470N mutations do not diminish 8a–8b binding; rather, it appears to remodel the binding pocket in a way that favors stabilization of these analogues. A general trend observed is that binding affinity toward the mutant PfCPSF3 increases with elongation of the alkyl spacer which was the inferred trend from the biological activity. In previously characterized systems, the PfCPSF3 double mutation (H36Y/D470N) was associated with reduced susceptibility to certain benzoxaborole chemotypes.^23^ The present molecular docking studies suggest that the rational hybridization of triazole-linked benzoxaborole and 4-aminoquinoline scaffold may confer enhanced adaptability to the altered pocket geometries, potentially through optimized steric complementarity or more favorable accommodation within induced-fit conformations to enhance binding affinity in a mutated environment. The mutation-tolerant binding profile positions compounds 8b–8e as promising leads for further optimization against the *P. falciparum* resistant strain.

**Table 5:** Induced Fit Docking (IFD) binding affinity of synthesized compounds on PfCPSF3-WT (3D7) and PfCPSF3-MT (W2)

Plasmodium target	Compound	Spacer (*n*)	Glide IFD score (kcal mol^−1^)	Glide docking score (kcal mol^−1^)	Glide XP score (kcal cal^−1^)
PfCPSF3-WT	8a	2	−1012.53	−9.43	−10.70
	8b	3	−1007.52	−7.16	−8.70
	8c	4	−1009.17	−8.17	−9.69
	8d	6	−1012.76	−8.11	−9.66
	8e	8	−1013.13	−8.15	−9.70
	cq^+^	—	−1003.22	−4.97	−6.55
	qn	—	−999.42	−5.17	−7.31
PfCPSF3-MT	8a	2	−1017.15	−8.24	−9.87
	8b	3	−1017.22	−7.91	−9.45
	8c	4	−1018.71	−9.25	−10.77
	8d	6	−1019.97	−9.12	−11.05
	8e	8	−1021.46	−10.14	−11.68
	cq^+^	—	−1012.33	−5.20	−6.78
	qn	—	−1006.58	−4.89	−7.03

### 2.7 Protein–ligand interactions revealed by MD simulations

The root-mean-square deviation (RMSD) profiles of PfCPSF3-WT (Fig. S2) and PfCPSF3-MT (Fig. S3) in complex with 8a–8e were analyzed to assess the stability of the systems over 100 ns molecular dynamics (MD) simulations. The protein RMSD (blue line) remains relatively stable throughout the simulation, exhibiting only a slight upward trend without significant fluctuations. This behavior indicates that the protein structure undergoes minimal deviation from its initial conformation, reflecting overall structural stability. In comparison, the ligand RMSD (red line) is maintains a general stability but displays greater variability than the protein. This suggests some degree of conformational flexibility exists within the binding site while still maintaining consistent interactions with the protein throughout the simulation.

**Fig. 4:**
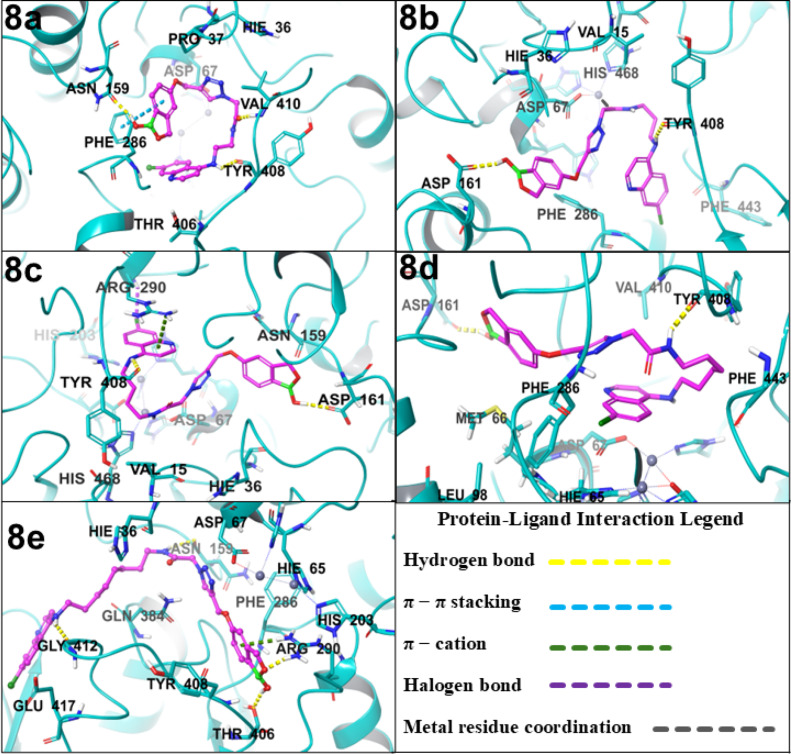
Three-dimensional binding modes and interaction profiles of the top induced-fit docking (IFD) poses of benzoxaborole-4-aminoquinoline hybrids (8a–8e) within the catalytic pocket of wild-type *Plasmodium falciparum* CPSF3 (PfCPSF3-WT), highlighting key hydrogen-bonding, π–π, π–cation, and Zn^2+^ coordination interactions with active-site residues.

**Fig. 5:**
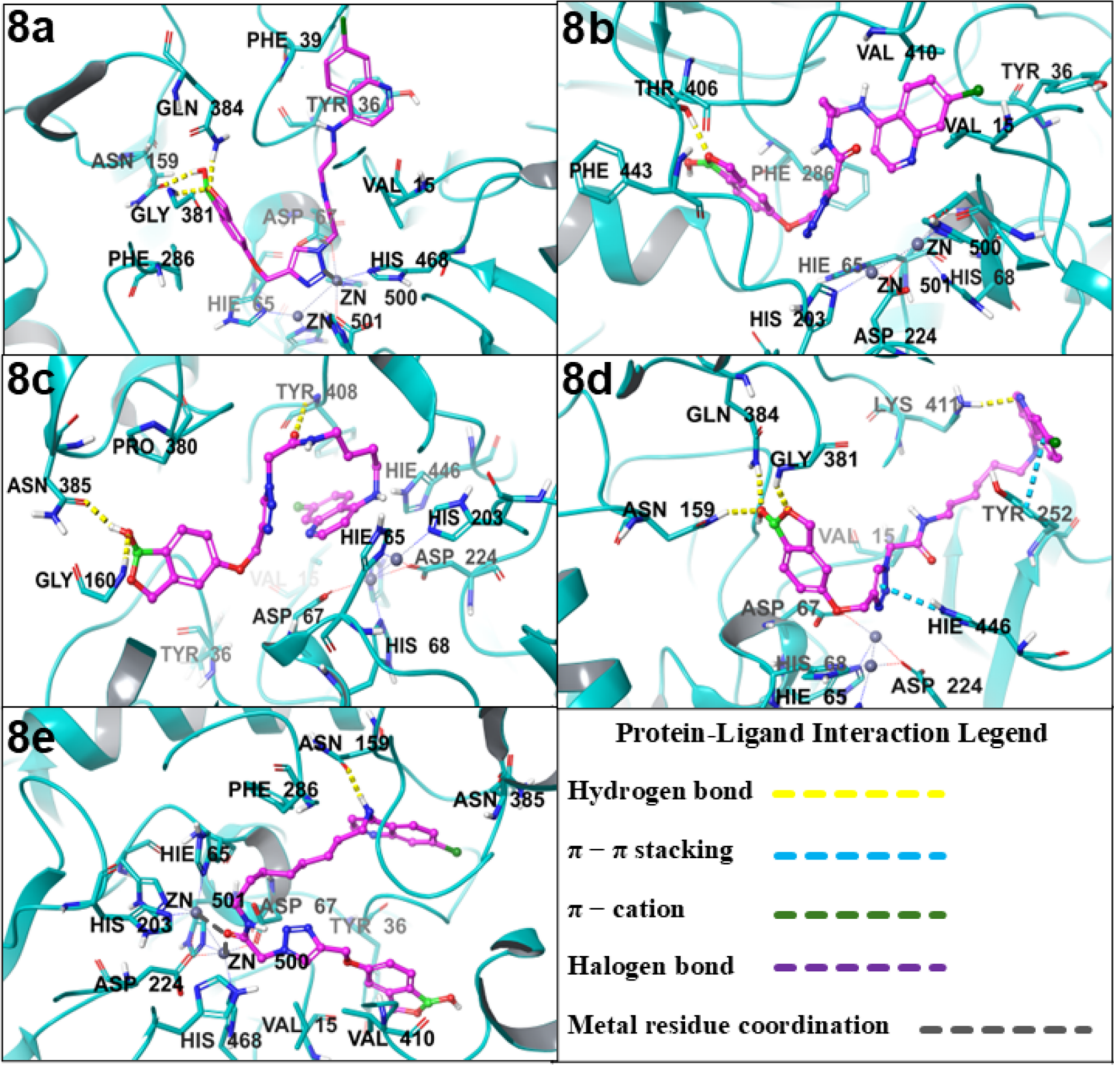
Three-dimensional binding modes and interaction profiles of the top induced-fit docking (IFD) poses of benzoxaborole-4-aminoquinoline hybrids (8a–8e) within the catalytic pocket of mutant *Plasmodium falciparum* CPSF3 (PfCPSF3-MT), highlighting key hydrogen-bonding, π–π, π–cation, and Zn^2+^ coordination interactions with active-site residues.

Protein–ligand interactions for wild-type and mutant PfCPSF3 complexes were monitored over 100 ns MD simulations, focusing on hydrogen bonds, hydrophobic contacts, water bridges, and ionic interactions. The analysis assessed the stability of binding pose and interactions identified during the induced-fit docking (Fig. 4 and 5) and revealed additional interactions not captured in the static conformation from the IFD. The various protein–ligand contacts captured over the simulation time frame are depicted using normalized stacked bar chart plots of the contact residues (Fig. S4 and S5) and 2D ligand–receptor interactions (Fig. S6 and S7).

#### 2.7.1 Protein–ligand interactions for 8a–8e-PfCPSF3-WT complexes

The predicted IFD binding modes of 8a–8e-PfCPSF3-WT complexes (Fig. 4) evaluated by MD simulations provided complementary dynamic insights (Fig. S6). All compounds exhibited stable binding, supported by conserved hydrogen bonding, π–π interactions, water-mediated contacts, and, in some cases, zinc ion coordination. Compound 8e exhibits a persistent interaction network, anchored by a hydrogen bond between the acetamide nitrogen and Asn159 (91%). Additional hydrogen bonds involve Glu249 (36%) and Tyr408 (21%) with the hydroxyl of the benzoxaborole fragment, Met66 (39%) with the triazole ring, Ala38 (28%) with the alkyl spacer nitrogen, and Gln384 (35%) with the acetamide oxygen. A π–π interaction between the triazole ring and Phe286 (78%) further stabilizes binding, indicating a cooperative network of polar and aromatic contacts.

The 8b-PfCPSF3-WT complex is characterized by strong metal-assisted stabilization, with near-complete coordination of the acetamide oxygen and triazole ring to the catalytic Zn^2+^ ions (99–100%). This is complemented by hydrogen bonds between Tyr408 (58%) and Val15 (85%) with the alkyl spacer amine and the acetamide nitrogen, respectively. Aromatic stabilization includes π–π stacking between His65 (81%) and the benzoxaborole ring and His446 (74%) with the triazole, while a water-mediated contact between the quinoline nitrogen and Phe443 (59%) further supports ligand positioning. Compound 8a maintains stable binding, anchored by hydrogen bonds between the hydroxyl of the benzoxaborole fragment and Asn159 (77%), and His446 (91%) with the acetamide oxygen. Additional stabilization arises from water-mediated contacts between the quinoline nitrogen and Thr406 (26%) and Pro284 (27%), along with π–π stacking with His36 (35%) and the triazole ring.

Furthermore, compound 8c exhibits an interaction network anchored by a hydrogen bond between Val15 (86%) and the acetamide nitrogen. Additional hydrogen bonds involve Asn159 (38%) with the hydroxyl of the benzoxaborole fragment and Asn165 (21%) with the oxaborole oxygen. In comparison, a water-mediated contact between the benzoxaborole hydroxyl group and Gly162 (23%) further supports ligand positioning. Compound 8d is stabilized by both direct and solvent-mediated interactions, including a hydrogen bond between Leu98 (54%) and the hydroxyl of the benzoxaborole fragment. Water-mediated hydrogen bonds involving Met66 (40%) and Asp67 (38%) with the triazole nitrogen further contribute to ligand orientation within the binding pocket. Overall, these findings indicate that 8a–8e binding within the PfCPSF3-WT active site is primarily governed by conserved hydrogen bonding interactions involving the benzoxaborole scaffold and surrounding residues, complemented by aromatic and, in one case, metal-coordination interactions that collectively stabilize the complexes.

#### 2.7.2 Protein–ligand interactions for 8a–8e-PfCPSF3-MT complexes

The IFD predicted binding modes of 8a–8e-PfCPSF3-MT complexes (Fig. 5) and were also evaluated with MD simulations in capturing protein-ligand interactions for the simulation time frame (Fig. S7). Compound 8e exhibits robust stabilization, anchored by hydrogen bonds between the alkyl spacer amine and Asn159 (54%) and the acetamide nitrogen with Asp67 (73%). The interaction is reinforced by coordination of the acetamide oxygen to the catalytic zinc ions (95% and 100%) and water-mediated contacts involving Asn385 with the quinoline nitrogen (30%) and the hydroxyl of the benzoxaborole scaffold (22%). In the 8d-PfCPSF3-MT complex, 8d maintains stability through a persistent (81%) hydrogen bond between Val15 and the acetamide nitrogen, supplemented by water-mediated interactions with the triazole and quinoline nitrogens and reinforced by π–π stacking (His65, Tyr408) and a cation–π interaction with the quinoline ring. Compound 8c displays a balanced interaction profile, with hydrogen bonding between Gly160 (59%) and the hydroxyl of the benzoxaborole fragment, while Asp67 (34%) interacts with the oxygen linker connecting the quinoline and triazole rings. These interactions are further supported by water-mediated contact by Met66 (38%) to the triazole nitrogen and π–π interactions involving the quinoline ring and triazole ring with Tyr408 (57%) and His65 (46%), respectively. Compound 8c, also exhibits notable zinc coordination with the quinoline nitrogen (61%) and acetamide oxygen (60%) further contributes to stabilization. In compound 8b, within the mutant active site, the acetamide group anchors the ligand through its nitrogen, forming hydrogen bonds with Val15 (35%) and Lys411 (24%), while the carbonyl oxygen interacts with Ala38 (24%). A water-mediated hydrogen bond between Lys411 (29%) and a triazole nitrogen further stabilizes ligand positioning. Additionally, the triazole ring coordinates to the catalytic zinc ion (39%), providing a transient but functionally relevant contribution to complex stability.

Compound 8a exhibits an interaction profile defined by strong hydrogen bonds involving the Asn159 and the hydroxyl of the benzoxaborole scaffold. In addition, Val15 (74%) forms a stable hydrogen bond with the acetamide nitrogen. Pronounced π–π stacking interactions are observed between His65 (92%) and the benzoxaborole benzene ring, as well as between His446 (88%) and the triazole ring, as well as a transient π–π interaction between Tyr36 (27%) and the quinoline moiety. Persistent zinc coordination is observed, with catalytic zinc ions interacting with nitrogen atoms of the triazole ring (84% and 98%), thereby reinforcing ligand anchoring within the active site.

#### 2.7.3 Protein–ligand interactions summary for PfCPSF3-MT and PfCPSF3-WT

In PfCPSF3-WT, compound 8b interacts predominantly through ionic contacts with zinc-coordinating residues, including His63, His65, Asp67, Asp68, His203, Asp224, and His468. In contrast, compounds 8a and 8c–8e are primarily stabilized by hydrophobic interactions, hydrogen bonding, and water bridges, with only limited ionic contributions (observed for 8c and 8d) or none (8a and 8e) (Fig. S4). In PfCPSF3-MT, however, compounds 8a–8c and 8e maintain persistent zinc coordination (Fig. S7) and exhibit strong ionic interactions with the zinc-coordinating residues, whereas 8d displays transient coordination associated with moderate ionic contacts (Fig. S5). Overall, the enhanced binding affinity of compounds 8a–8e in the mutant is likely driven by strengthened zinc-mediated interactions, together with an increase in π–π interactions relative to PfCPSF3-WT. Collectively, ligand stabilization in PfCPSF3-MT appears to arise from cooperative contributions of the benzoxaborole scaffold, the triazole-acetamide linker zinc coordination, and aromatic interactions, which together compensate for mutation-induced structural and energetic changes.

## 3 Conclusion

A structurally diverse library of twenty 4-aminoquinoline derivatives and benzoxaborole-4-aminoquinoline hybrids linked through a 1*H*-1,2,3-triazole spacer was synthesized and evaluated against CQ-sensitive (3D7) and CQ-resistant (W2) strains of *P. falciparum*. Stepwise optimization from azides to triazole-linked aldehydes, alcohols, and benzoxaborole hybrids led to a substantial enhancement in activity against parasite strains. Among the synthesized compounds, the benzoxaborole-4-aminoquinoline hybrids displayed the highest activity, particularly against the CQ-resistant W2 strain. Compound 8b emerged as the most potent compound among the synthesized hybrids, exhibiting superior activity to CQ and quinine against the resistant strain, and efficacy comparable to desethylamodiaquine. Several compounds showed low resistance indices, indicating a reduced susceptibility to established CQ-resistance mechanisms. Mechanistic investigations revealed that compound 8b binds monomeric heme more strongly than chloroquine under both physiological and acidic conditions, supporting inhibition of hemozoin formation as a primary mode of action. This mutation-tolerant binding, enabled by the complementary combination of the 4-aminoquinoline and benzoxaborole scaffolds, aligns with the experimental antiplasmodial data and supports the hypothesized mechanism of action involving both hemozoin inhibition and PfCPSF3 engagement, collectively validating benzoxaborole-4-aminoquinoline hybrids as a promising antimalarial chemotype against drug-resistant *P. falciparum*. Collectively, these findings identify benzoxaborole–4-aminoquinoline hybrids, particularly compound 8b, as promising antimalarial candidates with potential to combat drug-resistant *P. falciparum* and warrant further biological validation.

## Conflicts of interest

The authors declare that they have no known competing financial interests or personal relationships that could have influenced the work reported in this paper.

## Supplementary Material

RA-OLF-D6RA02824A-s001

## Data Availability

The data supporting this article have been included as part of the supplementary information (SI). Supplementary information is available. See DOI: https://doi.org/10.1039/d6ra02824a.
