# Supplementary material for: 4-Aminoquinoline derivatives and benzoxaborole-4-aminoquinoline hybrids: synthesis, antiplasmodial evaluation, heme-inhibition and in silico studies
Source: RSC Adv. 2026 Jul 8;16(36):38674–85. doi: 10.1039/d6ra02824a (PMC13344191; doi:10.1039/d6ra02824a)
Supplement: Supplementary file 1 [file RA-016-D6RA02824A-s001.pdf]

Supplementary Information (SI) for RSC Advances.
This journal is © The Royal Society of Chemistry 2026

4-Aminoquinoline derivatives and Benzoxaborole-4-Aminoquinoline Hybrids: Synthesis, Antiplasmodial evaluation, Heme-Inhibition and *In Silico* studies

Anu Saini[a], Sumit Kumar[a,b,c], Bruno Pradines[d,e,f,g], Océane Delandre[d,e,f,g], Joel Mosnier[d,e,f,g], Isabelle Fonta[d,e,f,g], Yukta Soni[a], Patrick Appiah-Kubi[i], Ashona Singh[i], Raghu Raj[h*], Vipan Kumar[a*]

[a]*Department of Chemistry, Guru Nanak Dev University, Amritsar, India*
[b] *Department of Biomedicine, University of Bergen, 5007 Bergen, Norway*
[c] *Department of Chemistry, University of Bergen, Allégaten 41, 5007 Bergen, Norway*
[d]*Unité Parasitologie et Entomologie, Departement Risques Infectieux, Institut de Recherche Biomédicale des Armées, Marseille, France*
[e]*Aix Marseille Univ, SSA, AP-HM, RITMES, Marseille, France*
[f]*IHU Méditerranée Infection, Marseille, France*
[g]*Centre National de Référence du Paludisme, Marseille, France*
[h]*PG Department of Chemistry, DAV College, Amritsar, India*
[i]*Department of Chemistry, University of Pretoria, Gauteng, South Africa*
*Corresponding Author vipan_org@yahoo.com (VK), raghusharma4@gmail.com (RR)

Experimental section

^{1}H NMR spectra were recorded in deuterochloroform ($CDCl_3$) andDMSO-d$_6$ with Bruker 500 (500 MHz) spectrometer using TMS as an internal standard. Chemical shift values are expressed as parts per million downfield from TMS and Jvalues are in hertz. Splitting patterns are indicated as s: singlet, d: doublet, t: triplet, m: multiplet, dd: double doublet, ddd: doublet of adoublet of a doublet, and br: broad peak. ^{13}C NMR spectra were recordedon a Bruker 500 (125 MHz) spectrometerin deuterochloroform ($CDCl_3$) and dimethylsulfoxide (DMSO-d$_6$) using TMS as internal standard. High resolution mass spectra were recordedon a Bruker-micrOTOF-Q II spectrometer.

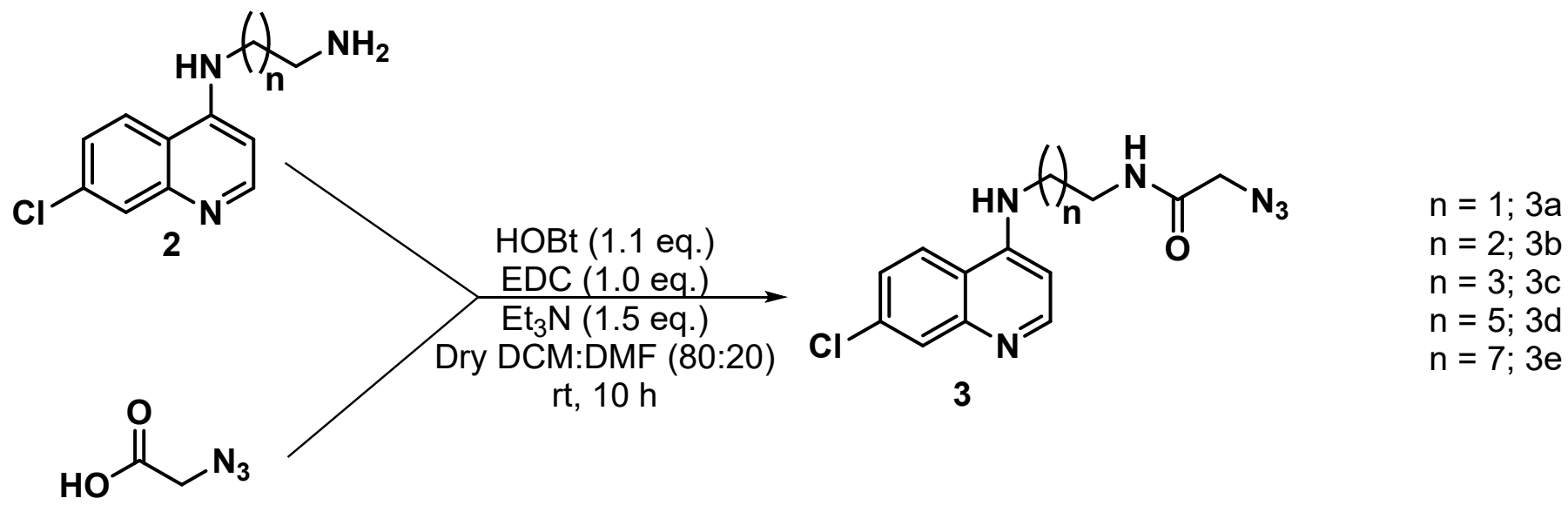

*1.1. General procedure for the synthesis of precursors **3a-e***. Example **3a**: To the stirred solution of diaminoquinoline **2a** (2 g, 1 mmol), prepared from 4,7-Dichloroquinoline and various diamines at 120 °C for overnight, and azidoacetic acid (0.911 g, 1 mmol) in Dry DCM (40 mL) and DMF (10 mL) mixture, add HOBt (1.34g, 1.1 mmol), EDC (1.72 g, 1.0 mmol) and Et_3N (1.95 ml, 1.5 mmol) at room temperature for 9-10 h yields precursor **3**. The completion of reaction was monitored by TLC. After usual workup with $CHCl_3$ and H_2O, $CHCl_3$ layers were

collected and dried over anhydrous Na_2SO_4 and further after solvent evaporation, crude product was obtained which was recrystallized using $CHCl_3$:MeOH mixture to get pure product **3** (2.50 g).

1.1.1 2-azido-N-(2-((7-chloroquinolin-4-yl)amino)ethyl)acetamide (**3a**): White Solid; Yield: 2.50 g, 91%; ^{1}H NMR (500 MHz, DMSO-d$_6$): δ 3.06-3.10 (m, 2H, -*N*-CH_2-), 3.22-3.26 (m, 2H, -*N*-CH_2-), 3.73 (s, 2H, -CH_2-), 6.47 (d, *J* = 5.5 Hz, 1H, H^2), 7.32 (t, *J* = 5.0 Hz, 1H, -NH- exchangeable with D_2O), 7.44 (dd, *J* = 2.0, 9.0 Hz, 1H, H^4), 7.78 (d, *J* = 2.0 Hz, 1H, H^5), 8.01 (t, *J* = 5.0 Hz, 1H, -NH- exchangeable with D_2O), 8.20 (d, *J* = 9.0 Hz, 1H, H^3), 8.38 (d, *J* = 5.5 Hz, 1H, H^1); ^{13}C NMR (125 MHz, DMSO- d$_6$): δ ppm = 39.1, 42.2, 51.0, 99.0, 117.9, 124.4, 124.5, 127.7, 133.6, 149.6, 150.3, 152.5, 162.4. HRMS Calculated for $C_{13}H_{13}ClN_6O$ [M+1] 305.0839 and [M+3] 307.0839 found 305.0850 and 307.0832; Anal. Calcd. (%) for: C, 51.24; H, 4.30; N, 27.58; Found: C, 51.10; H, 4.18; N, 27.69.

1.1.2 2-azido-N-(3-((7-chloroquinolin-4-yl)amino)propyl)acetamide (**3b**): White Solid; Yield: 2.48 g, 92%; ^{1}H NMR (500 MHz, DMSO-d$_6$): δ 1.90-1.93 (m, 2H, -CH_2-), 3.08-3.12 (m, 2H, -*N*-CH_2-), 3.25-3.29 (m, 2H, -*N*-CH_2-), 3.78 (s, 2H, -CH_2-), 6.49 (d, *J* = 5.5 Hz, 1H, H^2), 7.29 (t, *J* = 5.0 Hz, 1H, -NH- exchangeable with D_2O), 7.41 (dd, *J* = 2.0, 9.0 Hz, 1H, H^4), 7.77 (d, *J* = 2.0 Hz, 1H, H^5), 8.05 (t, *J* = 5.0 Hz, 1H, -NH- exchangeable with D_2O), 8.28 (d, *J* = 9.0 Hz, 1H, H^3), 8.35 (d, *J* = 5.5 Hz, 1H, H^1); ^{13}C NMR (125 MHz, DMSO-d$_6$): δ ppm = 28.1, 39.1, 42.2, 51.0, 99.0, 117.9, 124.4, 124.5, 127.7, 133.6, 149.6, 150.3, 152.5, 162.4. HRMS Calculated for $C_{14}H_{15}ClN_6O$ [M+1] 319.0996 and [M+3] 321.0996 found 319.1004 and 321.1009; Anal. Calcd. (%) for: C, 52.75; H, 4.74; N, 26.36; Found: C, 52.84; H, 4.65; N, 26.50.

1.1.3 2-azido-N-(4-((7-chloroquinolin-4-yl)amino)butyl)acetamide (**3c**): White Solid; Yield: 2.47 g, 93%; ^{1}H NMR (500 MHz, DMSO-d$_6$): δ 1.36-1.40 (m, 2H, -CH_2-), 1.60-1.63 (m, 2H, -CH_2-), 3.08-3.12 (m, 2H, -*N*-CH_2-), 3.22-3.26 (m, 2H, -*N*-CH_2-), 3.80 (s, 2H, -CH_2-), 6.48 (d, *J* = 5.5 Hz, 1H, H^2), 7.33 (t, *J* = 5.0 Hz, 1H, -NH- exchangeable with D_2O), 7.42 (dd, *J* = 2.0, 9.0 Hz, 1H, H^4), 7.76 (d, *J* = 2.0 Hz, 1H, H^5), 8.00 (t, *J* = 5.0 Hz, 1H, -NH- exchangeable with D_2O), 8.23 (d, *J* = 9.0 Hz, 1H, H^3), 8.37 (d, *J* = 5.5 Hz, 1H, H^1); ^{13}C NMR (125 MHz, DMSO-d$_6$): δ ppm = 25.8, 26.8, 38.8, 42.5, 51.2, 99.2, 117.7, 124.4, 124.6, 127.8, 133.8, 149.4, 150.0, 152.3, 162.6. HRMS Calculated for $C_{15}H_{17}ClN_6O$ [M+1] 333.1152 and [M+3] 335.1152 found 333.1141 and 335.1167; Anal. Calcd. (%) for: C, 54.14; H, 5.15; N, 25.25; Found: C, 54.30; H, 5.24; N, 25.16.

1.1.4 2-azido-N-(6-((7-chloroquinolin-4-yl)amino)hexyl)acetamide (**3d**): White Solid; Yield: 2.49 g, 96%; ^{1}H NMR (500 MHz, DMSO-d$_6$): δ 1.33-1.42 (m, 6H, 3×-CH_2-), 1.61-1.65 (m, 2H, -CH_2-), 3.05-3.09 (m, 2H, -*N*-CH_2-), 3.21-3.25 (m, 2H, -*N*-CH_2-), 3.79 (s, 2H, -CH_2-), 6.48 (d, *J* = 5.5 Hz, 1H, H^2), 7.29 (t, *J* = 5.0 Hz, 1H, -NH- exchangeable with D_2O), 7.42 (dd, *J* = 2.0, 9.0 Hz, 1H, H^4), 7.80 (d, *J* = 2.0 Hz, 1H, H^5), 8.05 (t, *J* = 5.0 Hz, 1H, -NH- exchangeable with D_2O), 8.23 (d, *J* = 9.0 Hz, 1H, H^3), 8.38 (d, *J* = 5.5 Hz, 1H, H^1); ^{13}C NMR (125 MHz, DMSO-d$_6$): δ

ppm = 26.0, 26.8, 27.9, 29.3, 38.7, 42.2, 51.1, 99.1, 117.9, 124.4, 124.5, 127.9, 133.6, 149.1, 150.5, 152.1, 163.6. HRMS Calculated for $C_{17}H_{21}ClN_6O$ [M+1] 361.1465 and [M+3] 363.1465 found 361.1473 and 363.1479; Anal. Calcd. (%) for: C, 56.59; H, 5.87; N, 23.29; Found: C, 56.43; H, 5.77; N, 25.38.

1.1.5 2-azido-N-(8-((7-chloroquinolin-4-yl)amino)octyl)acetamide (**3e**): White Solid; Yield: 2.39 g, 94%; ^1H NMR (500 MHz, DMSO-d$_6$): δ 1.25-1.42 (m, 10H, 5×-CH$_2$-), 1.64-1.67 (m, 2H, -CH$_2$-), 3.05-3.09 (m, 2H, -*N*-CH$_2$-), 3.23-3.27 (m, 2H, -*N*-CH$_2$-), 3.78 (s, 2H, -CH$_2$-), 6.45 (d, J = 5.5 Hz, 1H, H^2), 7.28 (t, J = 5.0 Hz, 1H, -NH- exchangeable with D_2O), 7.43 (dd, J = 2.0, 9.0 Hz, 1H, H^4), 7.77 (d, J = 2.0 Hz, 1H, H^5), 8.06 (t, J = 5.0 Hz, 1H, -NH- exchangeable with D_2O), 8.27 (d, J = 9.0 Hz, 1H, H^3), 8.38 (d, J = 5.5 Hz, 1H, H^1); ^{13}C NMR (125 MHz, DMSO-d$_6$): δ ppm = 26.7, 27.0, 28.2, 29.1, 29.3, 31.2, 39.0, 42.8, 51.2, 99.2, 117.9, 124.4, 124.5, 127.8, 133.8, 149.5, 150.5, 152.3, 162.7. HRMS Calculated for $C_{19}H_{25}ClN_6O$ [M+1] 389.1778 and [M+3] 391.1778 found 389.1788 and 391.1762; Anal. Calcd. (%) for: C, 58.68; H, 6.48; N, 21.61; Found: C, 58.80; H, 6.39; N, 21.50.

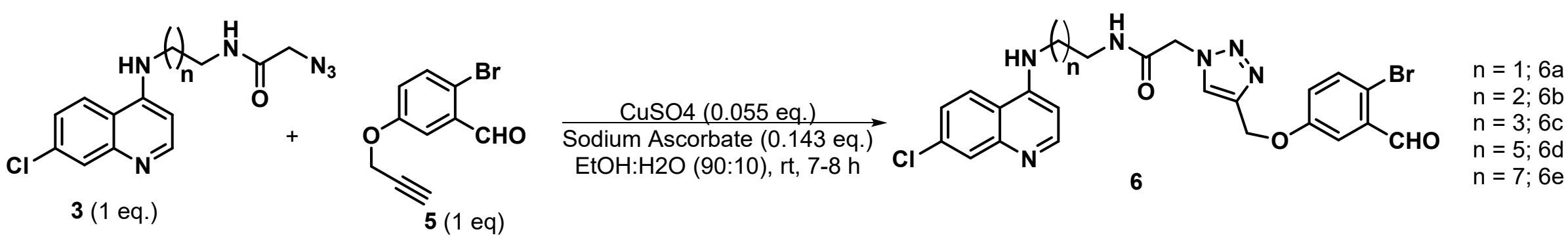

*1.2. General procedure for the synthesis of precursors **6a-e***. Example **6a**: The synthesized precursors **3a** (1.5 g, 1 mmol) were subjected to Cu-mediated click chemistry reaction with *O*-propargylated-2-bromobenzaldehyde **5** (1.17 g, 1 mmol) in EtOH (45 mL) and H_2O (5 mL) mixture in presence of $CuSO_4{\cdot}5H_2O$ (0.043 g, 0.055 mmol) and sodium ascorbate (0.139g, 0.143 mmol). The reaction mixture was stirred at room temperature for 7–8 h and progress of reaction was monitored *via* TLC. After the usual workup in $CHCl_3$ and H_2O, organic layers were collected, combined, and dried over anhydrous sodium sulphate and evaporated to give crude product which was further purified through column chromatography.

1.2.1. 2-(4-((4-bromo-3-formylphenoxy)methyl)-1H-1,2,3-triazol-1-yl)-N-(2-((7-chloroquinolin-4-yl)amino)ethyl)acetamide (**6a**): White Solid; Yield: 2.40 g, 90%; ^1H NMR (500 MHz, $CDCl_3$): δ 3.40 (s, 4H, 2×-N-CH$_2$-), 5.14 (s, 2H, -CH$_2$-), 5.26 (s, 2H, -CH$_2$-), 6.57 (d, J = 5.5 Hz, 1H, H^2), 7.33 (dd, J = 3.0, 8.5 Hz, 1H, H^8), 7.46-7.48 (m, 2H, -NH- exchangeable with D_2O + H^4), 7.50 (d, J = 3.5 Hz, 1H, H^7), 7.71 (d, J = 9.0 Hz, 1H, H^9), 7.80 (d, J = 2.0 Hz, 1H, H^5), 8.20 (d, J = 8.5 Hz, 2H, triazole-H H^6 + H^3), 8.42 (d, J = 5.5 Hz, 1H, H^1), 8.57 (br s, 1H, -NH- exchangeable with D_2O), 10.18 (s, 1H, -CHO); ^{13}C NMR (125 MHz, DMSO-d$_6$): δ ppm = 37.9, 42.2, 52.1, 62.0, 99.1, 115.4, 117.0, 117.8, 123.6, 124.4, 124.7, 126.7, 127.6, 134.0, 134.2, 135.3, 142.2, 149.1, 150.6, 152.0, 158.2, 166.4, 191.8. HRMS Calculated for $C_{23}H_{20}BrClN_6O_3$ [M+1]

543.0469 and [M+3] 545.0469 found 543.0459 and 545.0477; Anal. Calcd. (%) for: C, 50.80; H, 3.71; N, 15.45; Found: C, 50.92; H, 3.62; N, 15.59.

1.2.2. 2-(4-((4-bromo-3-formylphenoxy)methyl)-1H-1,2,3-triazol-1-yl)-N-(3-((7-chloroquinolin-4-yl)amino)propyl)acetamide (**6b**): White Solid; Yield: 2.31 g, 88%; ^{1}H NMR (500 MHz, $CDCl_3$): δ 1.85-1.88 (m, 2H, -CH_2-), 3.12-3.15 (m, 2H, -N-CH_2-), 3.22-3.25 (m, 2H, -N-CH_2-), 5.12 (s, 2H, -CH_2-), 5.26 (s, 2H, –CH_2–), 6.55 (*d*, J = 5.5 Hz, 1H, H^2), 7.36 (dd, J = 3.0, 8.5 Hz, 1H, H^8), 7.46-7.49 (m, 2H, -NH- exchangeable with D_2O + H^4), 7.55 (d, J = 3.5 Hz, 1H, H^7), 7.70 (d, J = 9.0 Hz, 1H, H^9), 7.77 (d, J = 2 Hz, 1H, H^5), 8.19 (s, 1H, triazole-H, H^6), 8.21 (d, J = 8.5 Hz, 1H, H^3), 8.41 (d, J = 5.5 Hz, 1H, H^1), 8.55 (t, J = 5.5 Hz, 1H, -NH- exchangeable with D_2O), 10.07 (s, 1H,–CHO); ^{13}C NMR (125 MHz, DMSO-d_6): δ ppm = 28.7, 38.1, 42.1, 52.5, 62.1, 99.0, 115.0, 117.5, 117.9, 123.4, 124.2, 124.7, 126.8, 128.1, 134.1, 134.4, 135.5, 142.2, 149.0, 150.3, 152.5, 159.1, 165.8, 191.0. HRMS Calculated for $C_{24}H_{22}BrClN_6O_3$ [M+1] 557.0625 and [M+3] 559.0625 found 557.0633 and 559.0638; Anal. Calcd. (%) for: C, 51.68; H, 3.98; N, 15.07; Found: C, 50.51; H, 3.87; N, 15.17.

1.2.3. 2-(4-((4-bromo-3-formylphenoxy)methyl)-1H-1,2,3-triazol-1-yl)-N-(4-((7-chloroquinolin-4-yl)amino)butyl)acetamide (**6c**): White Solid; Yield: 2.19 g, 85%; ^{1}H NMR (500 MHz, $CDCl_3$): δ 1.56-1.60 (m, 2H, -CH_2-), 1.65-1.70 (m, 2H, -CH_2-), 3.16-3.19 (m, 2H, -N-CH_2-), 3.24-3.28 (m, 2H, -N-CH_2-), 5.09 (s, 2H, -CH_2-), 5.18 (s, 2H, –CH_2–), 6.48 (*d*, J = 5.5 Hz, 1H, H^2), 7.09 (dd, J = 3.0, 8.5 Hz, 1H, H^8), 7.42-7.45 (m, 2H, -NH- exchangeable with D_2O + H^4), 7.50 (d, J = 3.5 Hz, 1H, H^7), 7.72 (d, J = 9.0 Hz, 1H, H^9), 7.80 (d, J = 2 Hz, 1H, H^5), 8.18 (s, 1H, triazole-H, H^6), 8.22 (d, J = 8.5 Hz, 2H, H^3), 8.42 (d, J = 5.5 Hz, 1H, H^1), 8.56 (t, J = 5.5 Hz, 1H, -NH- exchangeable with D_2O), 10.15 (s, 1H,–CHO); ^{13}C NMR (125 MHz, DMSO-d_6): δ ppm = 25.9, 27.0, 37.0, 42.1, 52.4, 62.3, 99.0, 115.3, 117.4, 117.9, 123.5, 124.6, 124.9, 126.4, 127.4, 134.0, 134.3, 135.1, 142.2, 149.6, 150.1, 152.3, 158.1, 165.9, 191.2. HRMS Calculated for $C_{25}H_{24}BrClN_6O_3$ [M+1] 571.0782 and [M+3] 573.0782 found 571.0769 and 573.0790; Anal. Calcd. (%) for: C, 52.51; H, 4.23; N, 14.70; Found: C, 52.61; H, 4.38; N, 14.83.

1.2.4. 2-(4-((4-bromo-3-formylphenoxy)methyl)-1H-1,2,3-triazol-1-yl)-N-(6-((7-chloroquinolin-4-yl)amino)hexyl)acetamide (**6d**): White Solid; Yield: 2.20 g, 88%; ^{1}H NMR (500 MHz, $CDCl_3$): δ 1.33-1.38 (m, 4H, 2×-CH_2-), 1.44-1.46 (m, 2H, -CH_2-), 1.63-1.66 (m, 2H, -CH_2-), 3.09-3.13 (m, 2H, -N-CH_2-), 3.22-3.26 (m, 2H, -N-CH_2-), 5.11 (s, 2H, -CH_2-), 5.24 (s, 2H, –CH_2–), 6.44 (*d*, J = 5.5 Hz, 1H, H^2), 7.30 (dd, J = 3.0, 8.5 Hz, 1H, H^8), 7.35 (t, J = 5.5 Hz, 1H, -NH- exchangeable with D_2O), 7.42 (dd, J = 2.0, 9.0 Hz, 1H, H^4), 7.47 (d, J = 3.5 Hz, 1H, H^7), 7.67 (d, J = 9.0 Hz, 1H, H^9), 7.78 (d, J = 1.5 Hz, 1H, H^5), 8.20 (s, 1H, triazole-H, H^6), 8.28 (d, J = 9.0 Hz, 1H, H^3), 8.37-8.39 (m, 2H, H^1 +-NH- exchangeable with D_2O), 10.15 (s, 1H,–CHO); ^{13}C NMR (125 MHz, DMSO-d_6): δ ppm = 26.6, 26.7, 28.1, 29.3, 39.2, 42.8, 52.1, 62.0, 99.3, 115.3, 117.0, 117.8, 123.5, 124.4, 124.6, 126.7, 127.6, 133.9, 134.1, 135.3, 142.2, 149.1, 150.7, 152.0, 158.1, 165.5, 191.7. HRMS Calculated for $C_{27}H_{28}BrClN_6O_3$ [M+1] 599.1095 and [M+3]

601.1095 found 599.1110 and 601.1086; Anal. Calcd. (%) for: C, 54.06; H, 4.70; N, 14.01; Found: C, 54.15; H, 4.84; N, 14.19.

1.2.5. 2-(4-((4-bromo-3-formylphenoxy)methyl)-1H-1,2,3-triazol-1-yl)-N-(8-((7-chloroquinolin-4-yl)amino)octyl)acetamide (**6e**): White Solid; Yield: 2.15 g, 89%; ^{1}H NMR (500 MHz, $CDCl_3$): δ 1.32-1.48 (m, 10H, 5×-CH_2-), 1.63-1.67 (m, 2H, -CH_2-), 3.06-3.10 (m, 2H, -*N*-CH_2-), 3.20-3.25 (m, 2H, -N-CH_2-), 5.08 (s, 2H, -CH_2-), 5.23 (s, 2H, –CH_2–), 6.46 (*d*, *J* = 5.5 Hz, 1H, H^2), 7.29 (dd, *J* = 3, 8.5 Hz, 1H, H^8), 7.33 (t, *J* = 5.5 Hz, 1H, -NH- exchangeable with D_2O), 7.42 (dd, *J* = 2.0, 9.0 Hz, 1H, H^4), 7.48 (d, *J* = 3.0 Hz, 1H, H^7), 7.66 (d, *J* = 9.0 Hz, 1H, H^9), 7.80 (d, *J* = 2.0 Hz, 1H, H^5), 8.21 (s, 1H, triazole-H, H^6), 8.24 (d, *J* = 9.0 Hz, 1H, H^3), 8.35-8.38 (m, 2H, H^1 +-NH- exchangeable with D_2O), 10.10 (s, 1H,–CHO);^{13}C NMR (125 MHz, DMSO-d_6): δ ppm = 26.6, 27.1, 28.4, 29.1, 29.2, 30.9, 39.1, 43.0, 52.1, 62.0, 98.6, 114.9, 117.4, 118.1, 123.9, 124.7, 125.2, 126.3 128.0, 133.8, 134.1, 135.4, 142.3, 150.2, 151.0, 152.8, 158.0, 166.4, 191.6. HRMS Calculated for $C_{29}H_{32}BrClN_6O_3$ [M+1] 627.1408 and [M+3] 629.1408 found 627.1420 and 627.1399; Anal. Calcd. (%) for: C, 55.47; H, 5.14; N, 13.38; Found: C, 55.33; H, 5.04; N, 13.54.

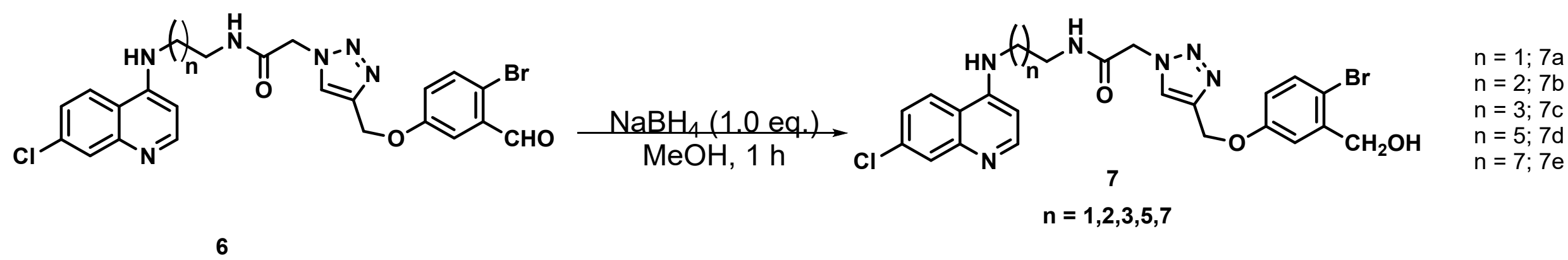

*1.3. General procedure for the synthesis of compounds 7**a-e***. Example **7a**: To the stirred solution of **4** (200 mg, 1 mmol) in methanol, add slowly $NaBH_4$ powder (13 mg, 1 mmol) at 0°C-rt. Then reaction mixture was allowed to stir for 1 h at room temperature. Reduce the volume of reaction mixture at low temperature under reduced pressure. After workup with $CHCl_3$:H_2O, collected organic layers were dried and evaporated to give white precipitate. Wash precipitates firstly with water and then with water/methanol mixture, and finally dried to give pure product **7a** (186 mg).

1.3.1. 2-(4-((4-bromo-3-(hydroxymethyl)phenoxy)methyl)-1H-1,2,3-triazol-1-yl)-N-(2-((7-chloroquinolin-4-yl)amino)ethyl)acetamide (**7a**): White Solid; Yield: 186 mg, 93%; ^{1}H NMR (500 MHz, $CDCl_3$): δ 3.40 (s, 4H, 2×-N-CH_2-), 4.47 (s, 2H, -CH_2-), 5.14 (s, 2H, -CH_2-), 5.16 (s, 2H, -CH_2-), 5.46 (br s, 1H, -NH- exchangeable with D_2O), 6.56 (d, *J* = 5.5 Hz, 1H, H^2), 6.93 (dd, *J* = 3.0, 8.5 Hz, 1H, H^8), 7.20 (d, *J* = 3.0 Hz, 1H, H^7), 7.40 (br s, 1H, -NH- exchangeable with D_2O), 7.45-7.47 (m, 2H, H^4+ H^9), 7.79 (d, *J* = 2 Hz, 1H, H^5), 8.18 (s, 1H, triazole-H, H^6), 8.20 (d, *J* = 9.0 Hz, 1H, H^3), 8.41 (d, *J* = 5.5 Hz, 1H, H^1), 8.58 (br s, 1H, -O-H exchangeable with D_2O); ^{13}C NMR (125 MHz, DMSO-d_6): δ ppm = 37.9, 42.1, 52.1, 61.6, 62.9, 99.1, 111.8, 115.1, 115.2, 117.9, 124.4, 124.6, 126.5, 127.9, 133.0, 133.9, 142.6, 142.7, 149.5, 150.4, 152.4, 158.1, 166.4. HRMS Calculated for $C_{23}H_{22}BrClN_6O_3$ [M+1] 545.0625 and [M+3] 547.0625 found

545.0613 and 547.0634; Anal. Calcd. (%) for: C, 50.61; H, 4.06; N, 15.40; Found: C, 50.74; H, 4.16; N, 15.28.

1.3.2. 2-(4-((4-bromo-3-(hydroxymethyl)phenoxy)methyl)-1H-1,2,3-triazol-1-yl)-N-(3-((7-chloroquinolin-4-yl)amino)propyl)acetamide (**7b**): White Solid; Yield: 180 mg, 90%; ^{1}H NMR (500 MHz, DMSO-d_6): δ 1.86-1.88 (m, 2H, -CH_2-), 3.33-3.39 (m, 4H, 2×-*N*-CH_2-), 4.49 (s, 2H, -CH_2-), 5.11 (s, 2H, -CH_2-), 5.16 (s, 2H, -CH_2-), 5.45 (br s, 1H, -O-H exchangeable with D_2O), 6.57 (d, J = 5.5 Hz, 1H, H^2), 6.94 (dd, J = 3.0, 9.0 Hz, 1H, H^8), 7.20 (d, J = 3.0 Hz, 1H, H^7), 7.40 (t, J = 5.5 Hz, 1H, -NH- exchangeable with D_2O), 7.44-7.46 (m, 2H, H^4+ H^9), 7.77 (d, J = 2 Hz, 1H, H^5), 8.19 (s, 1H, triazole-H, H^6), 8.23 (d, J = 9.0 Hz, 1H, H^3), 8.42 (d, J = 5.5 Hz, 1H, H^1), 8.58 (t, J = 5.5 Hz,1H, -NH- exchangeable with D_2O); ^{13}C NMR (125 MHz, DMSO-d_6): δ ppm = 28.6, 38.1, 42.3, 52.0, 62.0, 62.9, 99.4, 111.7, 115.0, 115.8, 117.1, 124.3, 124.9, 126.2, 127.8, 132.7, 133.5, 142.6, 142.7, 149.3, 150.2, 152.1, 158.0, 165.9. HRMS Calculated for $C_{24}H_{24}BrClN_6O_3$ [M+1] 559.0782 and [M+3] 561.0782 found 559.0774 and 561.0793; Anal. Calcd. (%) for: C, 51.49; H, 4.32; N, 15.01; Found: C, 51.61; H, 4.49; N, 15.11.

1.3.3. 2-(4-((4-bromo-3-(hydroxymethyl)phenoxy)methyl)-1H-1,2,3-triazol-1-yl)-N-(4-((7-chloroquinolin-4-yl)amino)butyl)acetamide (**7c**): White Solid; Yield: 178 mg, 89%; ^{1}H NMR (500 MHz, DMSO-d_6): δ 1.55-1.58 (m, 2H, -CH_2-), 1.66-1.69 (m, 2H, -CH_2-), 3.15-3.19 (m, 2H, -*N*-CH_2-), 3.26-3.30 (m, 2H, -*N*-CH_2-), 4.46 (d, J = 5.0 Hz, 2H, -CH_2-), 5.09 (s, 2H, -CH_2-), 5.16 (s, 2H, -CH_2-), 5.46 (t, J = 5.0 Hz, 1H, -NH- exchangeable with D_2O), 6.47 (d, J = 5.5 Hz, 1H, H^2), 6.92 (dd, J = 3.0, 8.5 Hz, 1H, H^8), 7.19 (d, J = 3.0 Hz, 1H, H^7), 7.31 (t, J = 5.0 Hz, 1H, -NH- exchangeable with D_2O), 7.43-7.47 (m, 2H, H^4+ H^9), 7.77 (d, J = 2.0 Hz, 1H, H^5), 8.16 (s, 1H, triazole-H, H^6), 8.27 (d, J = 9.0 Hz, 1H, H^3), 8.37-8.40 (m, 2H, H^1 + -O-H exchangeable with D_2O); ^{13}C NMR (125 MHz, DMSO-d_6): δ ppm = 25.6, 27.0, 38.9, 42.4, 52.1, 61.6, 63.0, 99.1, 111.8, 115.1, 115.2, 117.9, 124.4, 124.5, 126.5, 127.9, 133.1, 133.8, 142.6, 142.7, 149.5, 150.5, 152.4, 158.1, 165.6. HRMS Calculated for $C_{25}H_{26}BrClN_6O_3$ [M+1] 573.0938 and [M+3] 575.0938 found 573.0929 and 575.0931; Anal. Calcd. (%) for: C, 52.32; H, 4.57; N, 14.64; Found: C, 52.20; H, 4.67; N, 14.48.

1.3.4. 2-(4-((4-bromo-3-(hydroxymethyl)phenoxy)methyl)-1H-1,2,3-triazol-1-yl)-N-(6-((7-chloroquinolin-4-yl)amino)hexyl)acetamide (**7d**): White Solid; Yield: 182 mg, 91%; ^{1}H NMR (500 MHz, DMSO-d_6): δ 1.33-1.38 (m, 4H, 2×-CH_2-), 1.42-1.45 (m, 2H, -CH_2-), 1.66-1.69 (m, 2H, -CH_2-), 3.15-3.20 (m, 2H, -N-CH_2-), 3.22-3.26 (m, 2H, -N-CH_2-), 4.48 (s, 2H, -CH_2-), 5.13 (s, 2H, -CH_2-), 5.16 (s, 2H, -CH_2-), 5.46 (br s, 1H, -NH- exchangeable with D_2O), 6.53 (d, J = 5.5 Hz, 1H, H^2), 6.91 (dd, J = 3.0, 9.0 Hz, 1H, H^8), 7.15 (d, J = 3.0 Hz, 1H, H^7), 7.33 (t, J = 5.5 Hz, 1H, -NH- exchangeable with D_2O), 7.43-7.46 (m, 2H, H^4+ H^9), 7.80 (d, J = 2.0 Hz, 1H, H^5), 8.20 (s, 1H, triazole-H, H^6), 8.21 (d, J = 9.0 Hz, 1H, H^3), 8.38-8.40 (m, 2H, H^1+-O-H exchangeable with D_2O); ^{13}C NMR (125 MHz, DMSO-d_6): δ ppm = 25.5, 26.2, 28.8, 29.6, 39.0, 42.2, 52.1, 61.9, 62.8, 99.1, 111.9, 115.1, 115.3, 117.9, 124.5, 124.6, 126.6, 127.9, 133.4, 133.9, 142.6, 142.8, 149.1, 150.4, 152.6, 158.3, 166.0. HRMS Calculated for $C_{27}H_{30}BrClN_6O_3$ [M+1]

601.1251 and [M+3] 603.1251 found 601.1261 and 603.1264; Anal. Calcd. (%) for: C, 53.88; H, 5.02; N, 13.96; Found: C, 53.77; H, 5.11; N, 13.86.

1.3.5. 2-(4-((4-bromo-3-(hydroxymethyl)phenoxy)methyl)-1H-1,2,3-triazol-1-yl)-N-(8-((7-chloroquinolin-4-yl)amino)octyl)acetamide (**7e**): White Solid; Yield: 188 mg, 94%; ^{1}H NMR (500 MHz, DMSO-d$_6$): δ 1.24-1.38 (m, 8H, 4×-CH$_2$-), 1.44-1.47 (m, 2H, -CH$_2$-), 1.64-1.67 (m, 2H, -CH$_2$-), 3.12-3.16 (m, 2H, -N-CH$_2$-), 3.22-3.26 (m, 2H, -N-CH$_2$-), 4.46 (s, 2H, -CH$_2$-), 5.11 (s, 2H, -CH$_2$-), 5.20 (s, 2H, -CH$_2$-), 5.47 (br s, 1H, -NH- exchangeable with D$_2$O), 6.47 (d, J = 5.5 Hz, 1H, H^2), 6.89 (dd, J = 3.0, 9.0 Hz, 1H, H^8), 7.15 (d, J = 3.0 Hz, 1H, H^7), 7.34 (t, J = 5.5 Hz, 1H, -NH- exchangeable with D$_2$O), 7.44-7.47 (m, 2H, H^4+ H^9), 7.77 (d, J = 2.0 Hz, 1H, H^5), 8.20 (s, 1H, triazole-H, H^6), 8.25 (d, J = 9.0 Hz, 1H, H^3), 8.37-8.39 (m, 2H, H^1+-O-H exchangeable with D$_2$O); ^{13}C NMR (125 MHz, DMSO-d$_6$): δ ppm = 26.2, 26.9, 28.2, 29.0, 29.3, 31.0, 39.1, 42.4, 52.0, 62.1, 63.0, 99.0, 111.8, 115.1, 115.2, 117.9, 124.4, 124.6, 126.8, 127.9, 133.2, 133.9, 142.5, 142.6, 149.4, 150.5, 152.1, 158.0, 165.6. HRMS Calculated for $C_{29}H_{34}BrClN_6O_3$ [M+1] 629.1564 and [M+3] 631.1564 found 629.1573 and 631.1554; Anal. Calcd. (%) for: C, 55.29; H, 5.44; N, 13.34; Found: C, 55.15; H, 5.53; N, 13.51.

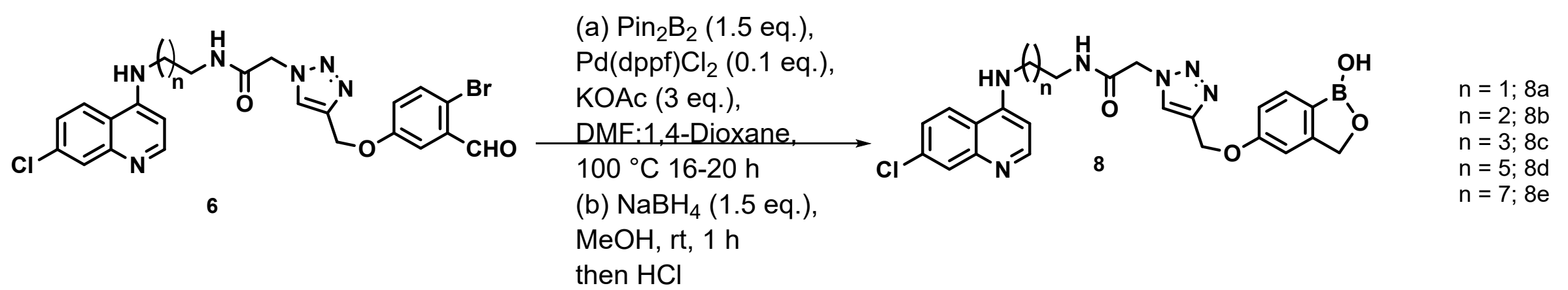

*1.4. General procedure for the synthesis of compounds **8a-e***. Example **8a**: After N_2 gas was bubbling through the solution of **6** (0.5 g, 1 mmol) in 1,4-dioxane (100 mL) for minimum of 15 min., add CH_3COOK (0.270 g, 3 mmol), Pd(dppf) Cl_2 (0.067g, 0.1 mmol) and bis(pinacolato)diboron (0.350 g, 1.5 mmol) and then allow to stir the reaction mixture for 16–20 h at 100 °C under N_2 environment. The reaction was monitored *via* TLC and reaction mixture was evaporated under reduced pressure to dryness. The residue was purified *via* column chromatography using 60–120 silica gel to afford boronic ester as light yellow waxy solid. Add slowly sodium borohydride (0.023 g, 1.5 mmol) in stirred solution of boronic ester in presence of methanol as solvent and then stir above reaction mixture for 1 h at room temperature which upon acidification with 1M HCl solution to pH 3 produces crude ppts. The precipitates were utilized for recrystallized using H_2O:MeOH mixture to afford pure product **8** (0.302 g).

1.4.1. N-(2-((7-chloroquinolin-4-yl)amino)ethyl)-2-(4-(((1-hydroxy-1,3-dihydrobenzo[c][1,2]oxaborol-5-yl)oxy)methyl)-1H-1,2,3-triazol-1-yl)acetamide (**8a**): White Solid; Yield: 0.302 g, 76%; ^{1}H NMR (500 MHz, DMSO-d$_6$): δ 3.06-3.10 (m, 2H, -N-CH$_2$-),

3.46-3.49 (m, 2H, -N-CH_2-), 4.44 (s, 2H, -CH_2-), 5.09 (s, 2H, -CH_2-), 5.17 (s, 2H, -CH_2-), 5.51 (br s, 1H, -NH-), 6.83 (d, J = 7.0 Hz, 1H, H^2), 6.92 (dd, J = 3.0, 8.5 Hz, 1H, H^8), 7.14 (d, J = 3.0 Hz, 1H, H^7), 7.45 (d, J = 8.5 Hz, 1H, H^9), 7.77 (dd, J = 2.0, 9.0 Hz, 1H, H^4), 8.00 (d, J = 2.0 Hz, 1H, H^5), 8.21 (s, 1H, triazole-H, H^6), 8.42 (t, J = 5.5 Hz, 1H, -NH- exchangeable with D_2O), 8.54 (d, J = 7.0 Hz, 1H, H^1), 8.60 (d, J = 9.0 Hz, 1H, H^3), 9.50 (br s, 1H, -OH); ^{13}C NMR (125 MHz, DMSO-d_6): δ ppm = 39.1, 43.4, 52.1, 61.6, 62.9, 98.9, 111.8, 115.0, 115.1, 115.9, 119.7, 126.2, 126.5, 126.9, 133.0, 138.0, 139.4, 142.5, 142.7, 143.4, 155.4, 158.0, 165.6. HRMS Calculated for $C_{23}H_{22}BClN_6O_4$ [M+1] 493.1484 and [M+3] 495.1484 found 493.1476 and 495.1470; Anal. Calcd. (%) for: C, 56.07; H, 4.50; N, 17.06; Found: C, 56.19; H, 4.34; N, 16.95.

1.4.2. N-(3-((7-chloroquinolin-4-yl)amino)propyl)-2-(4-(((1-hydroxy-1,3-dihydrobenzo[c][1,2]oxaborol-5-yl)oxy)methyl)-1H-1,2,3-triazol-1-yl)acetamide (**8b**): White Solid; Yield: 0.280 g, 70%; ^1H NMR (500 MHz, DMSO-d_6): δ 1.86-1.89 (m, 2H, -CH_2-), 3.23-3.27 (m, 2H, -N-CH_2-), 3.56-3.60 (m, 2H, -N-CH_2-), 4.45 (s, 2H, -CH_2-), 5.16 (s, 4H, 2×-CH_2-), 5.48 (br s, 1H, -NH- exchangeable with D_2O), 6.88 (d, J = 7.0 Hz, 1H, H^2), 6.91 (d, J = 8.5 Hz, 1H, H^9), 7.18 (d, J = 2.5 Hz, 1H, H^7), 7.44 (dd, J = 3.5, 8.5 Hz, 1H, H^8), 7.77 (dd, J = 2.0, 9.0 Hz, 1H, H^4), 8.03 (d, J = 2.0 Hz, 1H, H^5), 8.20 (s, 1H, triazole-H, H^6), 8.54 (d, J = 6.0 Hz, 1H, H^1), 8.63-8.71 (m, 2H, -NH- exchangeable with D_2O + H^3), 9.61 (br s, 1H, -OH); ^{13}C NMR (125 MHz, DMSO-d_6): δ ppm = 27.8, 36.8, 41.2, 52.2, 61.7, 62.9, 99.0, 111.8, 115.1, 115.2, 115.9, 119.6, 126.3, 126.6, 127.2, 133.1, 138.3, 139.1, 142.6, 142.7, 143.3, 155.8, 158.1, 165.9. HRMS Calculated for $C_{24}H_{24}BClN_6O_4$ [M+1] 507.1641 and [M+3] 509.1641 found 507.1628 and 509.1633; Anal. Calcd. (%) for: C, 56.88; H, 4.77; N, 16.58; Found: C, 56.99; H, 4.86; N, 16.67.

1.4.3. N-(4-((7-chloroquinolin-4-yl)amino)butyl)-2-(4-(((1-hydroxy-1,3-dihydrobenzo[c][1,2]oxaborol-5-yl)oxy)methyl)-1H-1,2,3-triazol-1-yl)acetamide (**8c**): White Solid; Yield: 0.265 g, 66%; ^1H NMR (500 MHz, DMSO-d_6): δ 1.52-1.55 (m, 2H, -CH_2-), 1.63-1.66 (m, 2H, -CH_2-), 3.08-3.12 (m, 2H, -N-CH_2-), 3.51-3.54 (m, 2H, -N-CH_2-), 4.48 (s, 2H, -CH_2-), 5.12 (s, 2H, -CH_2-), 5.15 (s, 2H, -CH_2-), 5.46 (br s, 1H, -NH- exchangeable with D_2O), 6.88 (d, J = 7.0 Hz, 1H, H^2), 6.92 (dd, J = 3.0, 8.5 Hz, 1H, H^8), 7.13 (d, J = 3.0 Hz, 1H, H^7), 7.44 (d, J = 8.5 Hz, 1H, H^9), 7.79 (dd, J = 2.0, 9.0 Hz, 1H, H^4), 7.98 (d, J = 2.0 Hz, 1H, H^5), 8.19 (s, 1H, triazole-H, H^6), 8.43 (t, J = 5.5 Hz, 1H, -NH- exchangeable with D_2O), 8.51 (d, J = 7.0 Hz, 1H, H^1), 8.61 (d, J = 9.0 Hz, 1H, H^3), 9.31 (br s, 1H, -OH); ^{13}C NMR (125 MHz, DMSO-d_6): δ ppm = 26.1, 27.3, 39.0, 43.1, 52.2, 61.7, 62.7, 99.1, 111.7, 115.1, 115.2, 115.9, 119.8, 126.1, 126.6, 127.2, 133.0, 138.1, 139.7, 142.4, 142.7, 143.1, 155.2, 158.6, 166.2. HRMS Calculated for $C_{25}H_{26}BClN_6O_4$ [M+1] 521.1797 and [M+3] 523.1797 found 521.1790 and 523.1785; Anal. Calcd. (%) for: C, 57.66; H, 5.03; N, 16.14; Found: C, 57.57; H, 4.88; N, 16.25.

1.4.4. N-(6-((7-chloroquinolin-4-yl)amino)hexyl)-2-(4-(((1-hydroxy-1,3-dihydrobenzo[c][1,2]oxaborol-5-yl)oxy)methyl)-1H-1,2,3-triazol-1-yl)acetamide (**8d**): White Solid; Yield: 0.282 g, 71%; ^1H NMR (500 MHz, DMSO-d_6): δ 1.32-1.46 (m, 6H, 3×-CH_2-), 1.66-1.69 (m, 2H, -CH_2-), 3.09-3.12 (m, 2H, -N-CH_2-), 3.48-3.52 (m, 2H, -N-CH_2-), 4.45 (s, 2H,

-CH_2-), 5.10 (s, 2H, -CH_2-), 5.15 (s, 2H, -CH_2-), 5.47 (br s, 1H, -NH- exchangeable with D_2O), 6.84 (d, J = 7.0 Hz, 1H, H^2), 6.90 (dd, J = 3.0, 8.5 Hz, 1H, H^8), 7.17 (d, J = 3.0 Hz, 1H, H^7), 7.44 (d, J = 8.5 Hz, 1H, H^9), 7.75 (dd, J = 2.0, 9.0 Hz, 1H, H^4), 7.99 (d, J = 1.5 Hz, 1H, H^5), 8.17 (s, 1H, triazole-H, H^6), 8.42 (t, J = 5.5 Hz, 1H, -NH- exchangeable with D_2O), 8.51 (d, J = 7.0 Hz, 1H, H^1), 8.60 (d, J = 9.0 Hz, 1H, H^3), 9.34 (br s, 1H, -OH); ^{13}C NMR (125 MHz, DMSO-d_6): δ ppm = 25.8, 26.4, 27.9, 29.2, 39.1, 43.4, 52.1, 61.6, 62.9, 98.9, 111.8, 115.0, 115.1, 115.9, 119.7, 126.2, 126.5, 127.0, 133.0, 138.1, 139.4, 142.5, 142.7, 143.4, 155.4, 158.0, 165.6. HRMS Calculated for $C_{27}H_{30}BClN_6O_4$ [M+1] 549.2110 and [M+3] 551.2100 found 549.2118 and 551.2120; Anal. Calcd. (%) for: C, 59.09; H, 5.51; N, 15.31; Found: C, 59.25; H, 5.38; N, 15.48.

1.4.5. N-(8-((7-chloroquinolin-4-yl)amino)octyl)-2-(4-(((1-hydroxy-1,3-dihydrobenzo[c][1,2]oxaborol-5-yl)oxy)methyl)-1H-1,2,3-triazol-1-yl)acetamide (**8e**): White Solid; Yield: 0.270 g, 68%; ^1H NMR (500 MHz, DMSO-d_6): δ 1.27-1.44 (m, 10H, 5×-CH_2-), 1.67-1.70 (m, 2H, -CH_2-), 3.08-3.11 (m, 2H, -N-CH_2-), 3.49-3.52 (m, 2H, -N-CH_2-), 4.47 (s, 2H, -CH_2-), 5.10 (s, 2H, -CH_2-), 5.16 (s, 2H, -CH_2-), 5.42 (br s, 1H, -NH- exchangeable with D_2O), 6.83 (d, J = 7.0 Hz, 1H, H^2), 6.94 (dd, J = 3.0, 8.5 Hz, 1H, H^8), 7.17 (d, J = 3.0 Hz, 1H, H^7), 7.45 (d, J = 8.5 Hz, 1H, H^9), 7.77 (dd, J = 2.0, 9.0 Hz, 1H, H^4), 8.00 (d, J = 2.0 Hz, 1H, H^5), 8.20 (s, 1H, triazole-H, H^6), 8.43 (t, J = 5.5 Hz, 1H, -NH- exchangeable with D_2O), 8.49 (d, J = 7.0 Hz, 1H, H^1), 8.56 (d, J = 9.0 Hz, 1H, H^3), 9.42 (br s, 1H, -OH); ^{13}C NMR (125 MHz, DMSO-d_6): δ ppm = 26.1, 27.0, 27.9, 29.2, 29.8, 31.5, 38.1, 42.7, 52.1, 61.7, 62.9, 99.0, 111.8, 115.0, 115.1, 115.8, 119.9, 126.3, 126.6, 127.3, 133.1, 137.9, 139.3, 142.5, 142.6, 143.6, 155.5, 158.1, 165.8. HRMS Calculated for $C_{29}H_{34}BClN_6O_4$ [M+1] 577.2423 and [M+3] 579.2423 found 577.2433 and 579.2414; Anal. Calcd. (%) for: C, 60.38; H, 5.94; N, 14.57; Found: C, 60.46; H, 6.07; N, 14.68.

1. ***Biological evaluation***

2.1. In vitro antiplasmodial assay

The two *P. falciparum* strains, the chloroquine-susceptible 3D7 (isolated in West Africa; obtained from MR4, VA, USA), and the chloroquine-resistant strain W2 (isolated in Indochina; obtained from MR4, VA, USA), were maintained in culture in RPMI 1640 (Invitrogen, Paisley, UK), supplemented with 10% human serum (Abcys S.A. Paris, France) and buffered with 25 µM HEPES and 25 µM $NaHCO_3$. Parasites were grown in A-positive human blood (Etablissement Français du Sang, Marseille, France) under controlled atmospheric conditions that consisted of

10% O_2, 5% CO_2 and 85% N_2 at 37 °C with a humidity of 95%. The two *P. falciparum* strains 3D7 and W2, were synchronized twice with sorbitol before use [1]. The clonality of these strains was every month by PCR genotyping of the polymorphic genetic markers *msp1* and *msp2* and microsatellite loci [2,3]. The compounds were re-suspended and then diluted RPMI-DMSO 5% (v/v) to obtain 11 final concentrations ranging from 0.78 nM to 10000 nM (final concentration of 0.5% of DMSO). For the *in vitro* assay, 25 µl of each concentration of benzoxaborole derivatives or antimalarial drugs used as comparator were aliquoted in one replicate with 200 µl of parasitized red blood cells (final parasitemia, 0.5%; final haematocrit, 1.5%) into 96-well plates. The plates were incubated for 72 h at 37 °C in controlled atmospheric conditions 5% CO_2, 10% O_2 and 75% N_2. After freezing and hemolysis, the HRP2 concentration (Histidine-rich protein 2) of the supernatants (different concentrations of drugs and control without drug), correlated with parasite growth, was evaluated at 72 h by the commercial test HRP2 ELISA (Malaria Ag Celisa, Cellabs PTY LTD, Brookvale, Australia). The optical density (OD) of each well was measured by spectrophotometry (Saphir 2, Tecan, Lyon, France). The concentration at which the drugs were able to inhibit 50% of parasite growth (IC_{50}) was calculated with the inhibitory sigmoid E_{max} model, with estimation of the IC_{50} through non-linear regression using a standard function of the R software (IC Estimator version 1.2). The antiplasmodial *in vitro* assay was performed five times for each product. The IC_{50} values represented the mean value (± standard deviation) calculated from five independent experiments.

2.2 ***Cytotoxicity Assay***

2.2.1 Cell Culturing

Cells were seeded into 96-well plates for 24 h to allow for attachment to wells prior to being treated with compounds. Prior to seeding into 96-well plates, cells were counted, and morphology was visually examined under an inverted bright field microscope. MCF-7 cells were cultured in DMEM media with 10% FBS, and 1% penicillin-streptomycin and MDA-MB-231 cells were cultured in DMEM, and Ham's F12 medium (3:1) supplemented with 10% FBS and 1% penicillin-streptomycin. Both were incubated at 37 °C and 5% carbon dioxide. All compounds were diluted in DMSO with a final concentration of 0.1% max (v/v).

2.2.2. MTT assay

Cells were seeded in 96 well plates at a density of 5000 cells per well in triplicate in media. After 24 hours, the test compounds, diluted in complete Dulbecco's media Eagle's medium (DMEM) were added to each well. Cells were treated with a range of different concentrations of drug (1, 5, 10, 20, 50, 100μM) for 24 hours at 37°C and 5% carbon dioxide. Subsequently, sterile 5μl of 5 mg/mL MTT (Sigma-Aldrich) dissolved in PBS was added to each well and incubated with cells for 2 hours. Solubilisation solution (10% SDS, 10mM HCl) of equal volume to the wells was then added to each well, which was incubated with cells for 16 hours at 37 °C [4,5]. The optical density of each well was read at 570 nm using a microtiter plate reader (Thermo Fisher Scientific Multiskan GO Microplate Reader, SkanIt™ software)

2.2.3. Statistical analysis

The statistical analysis was performed using Excel®, and IC_{50} values were estimated using Graphpad Prism5 software (Hearne Scientific Software) and the statistical significance was determined by one-way ANOVA. Two biological repeats were performed for each compound in triplicates (technical repeats). Additionally, Z-score was calculated for each plate, where a Z-score of >0.6 was considered technically sound with minimal standard deviation.

2. **Scanned ^{1}H and ^{13}C NMR spectra of representative compounds**:

^{1}H NMR of Compound 3e:

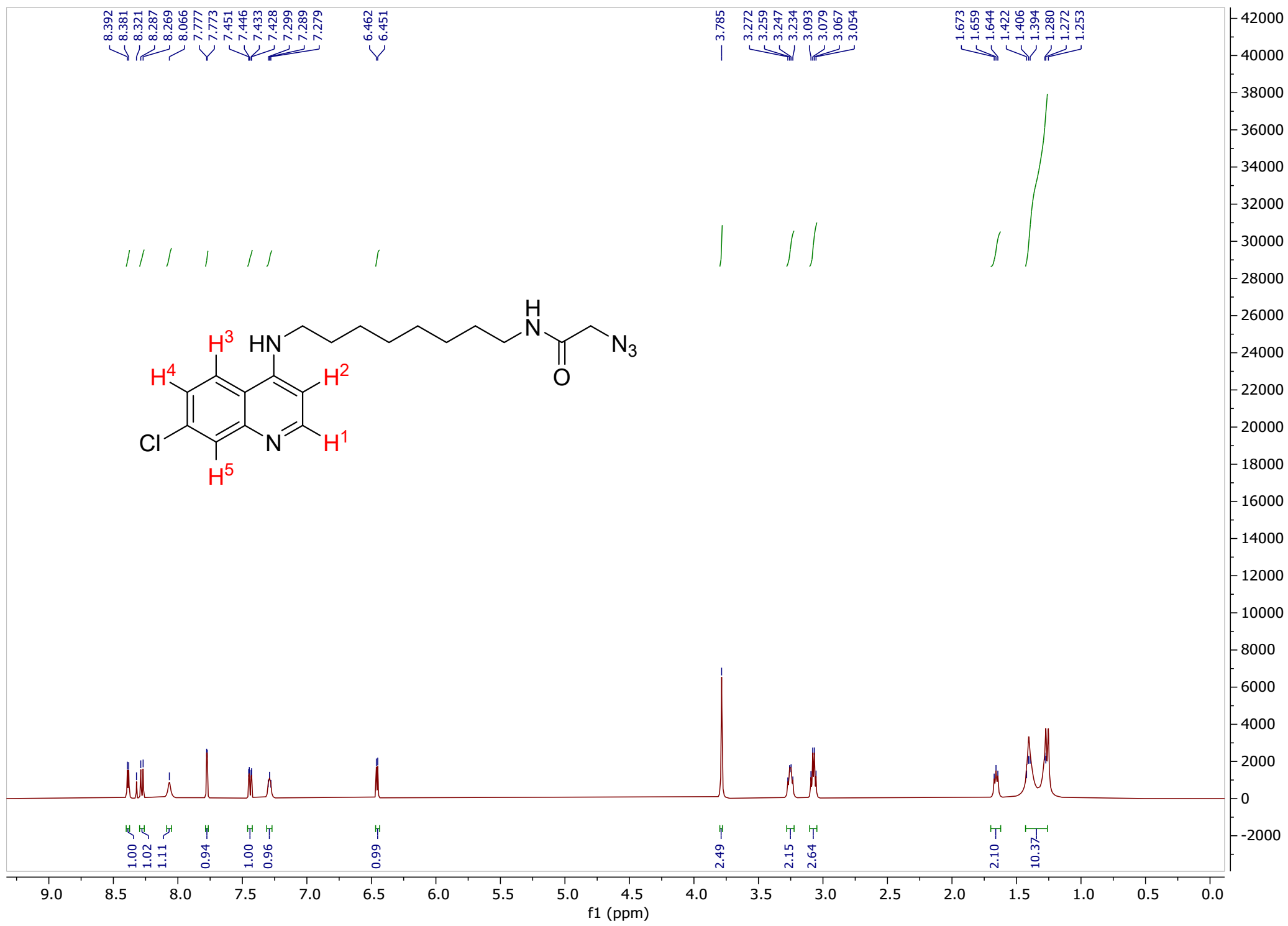

^{13}C NMR of Compound 3e:

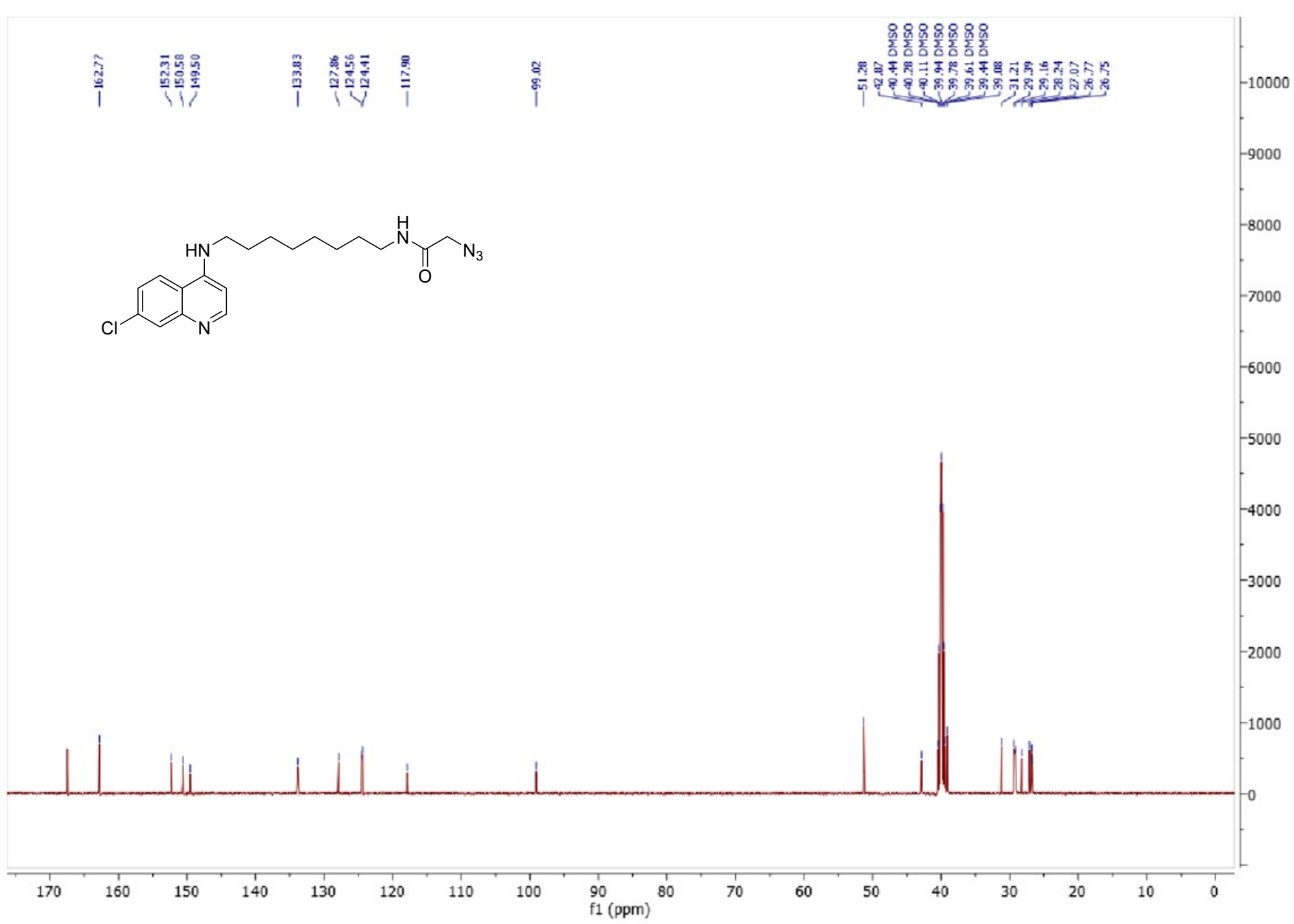

^{1}H NMR of Compound 4a:

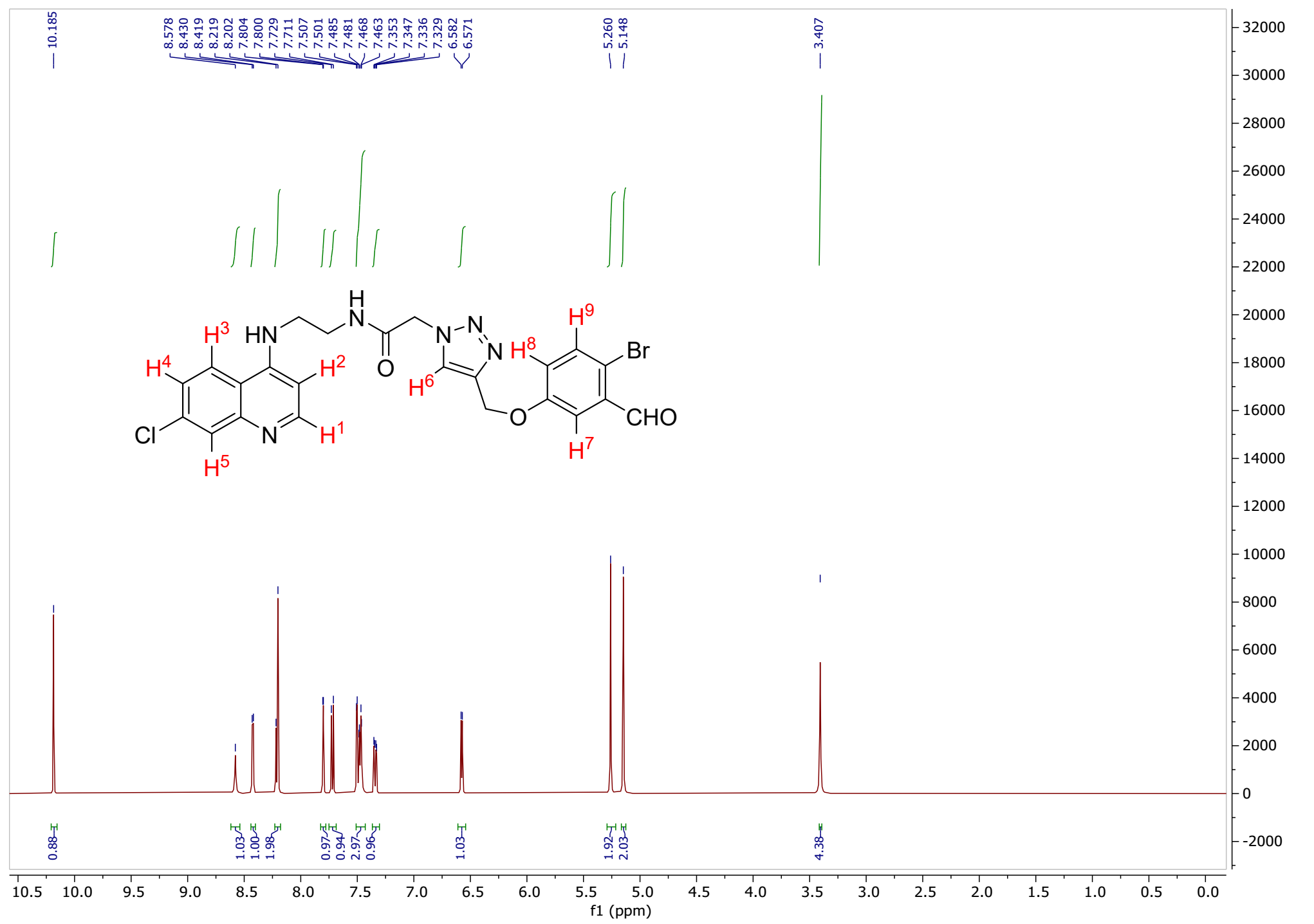

^{13}C NMR of Compound 4a:

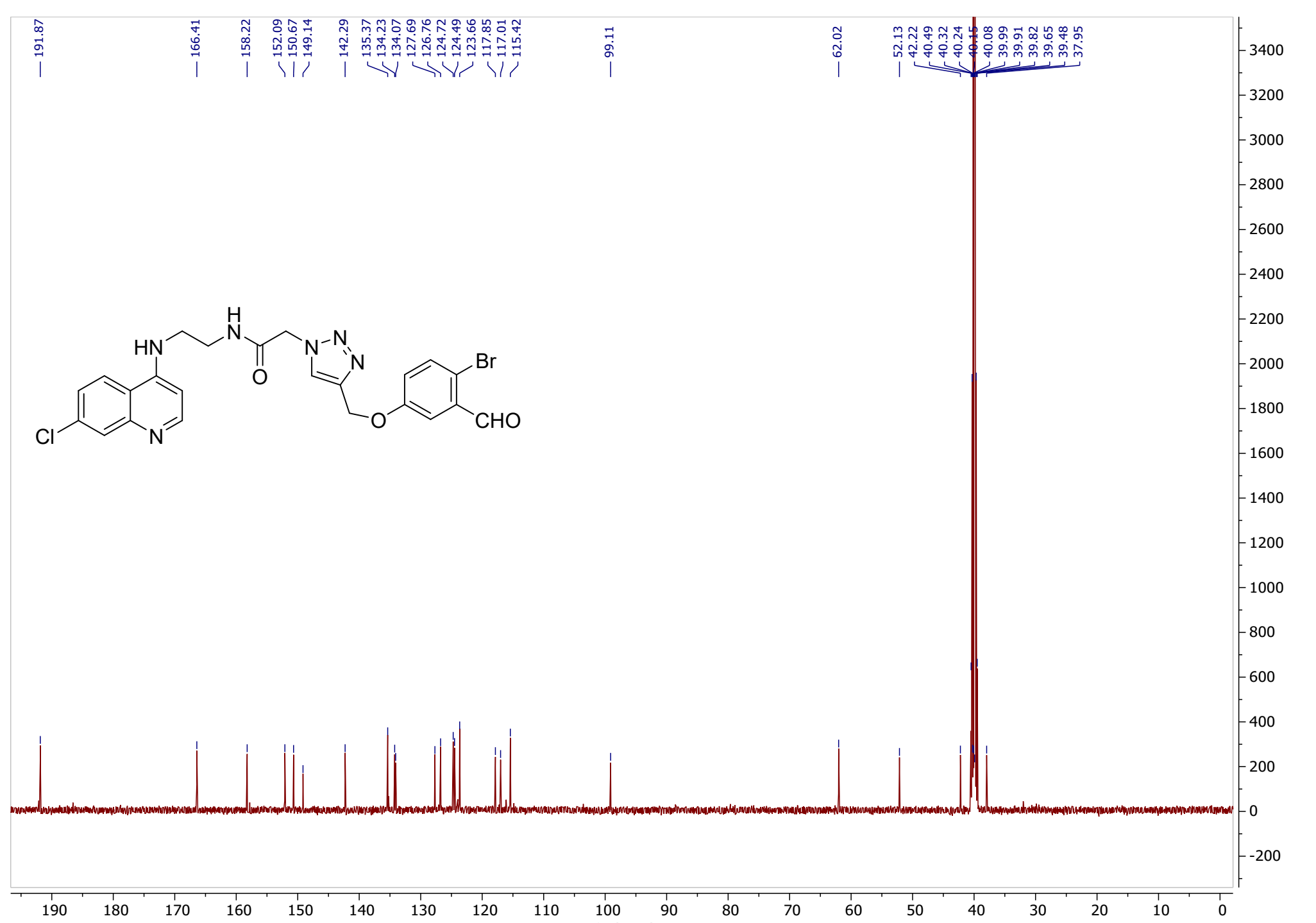

^{1}H NMR of Compound 4d:

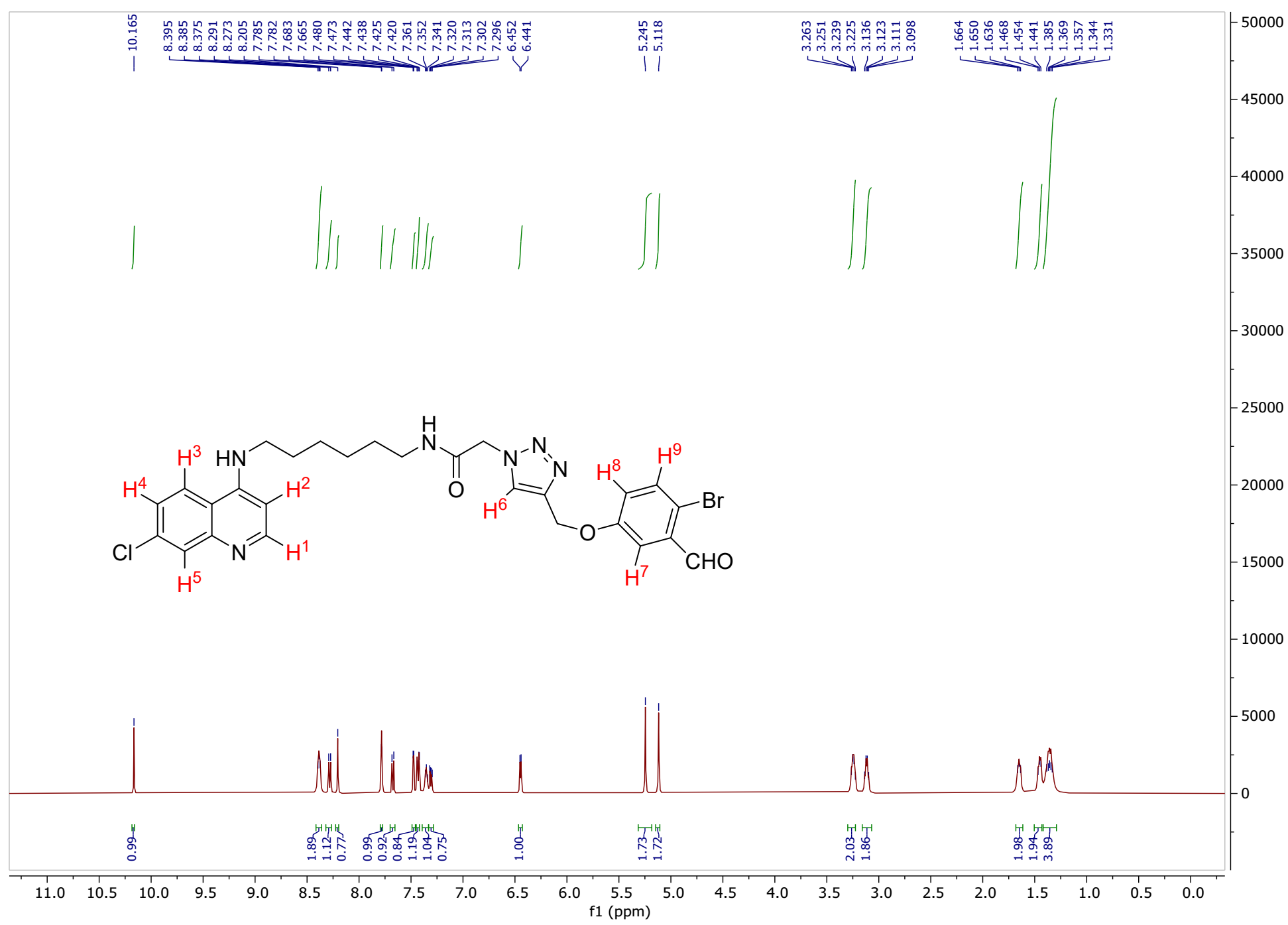

^{13}C NMR of Compound 4d:

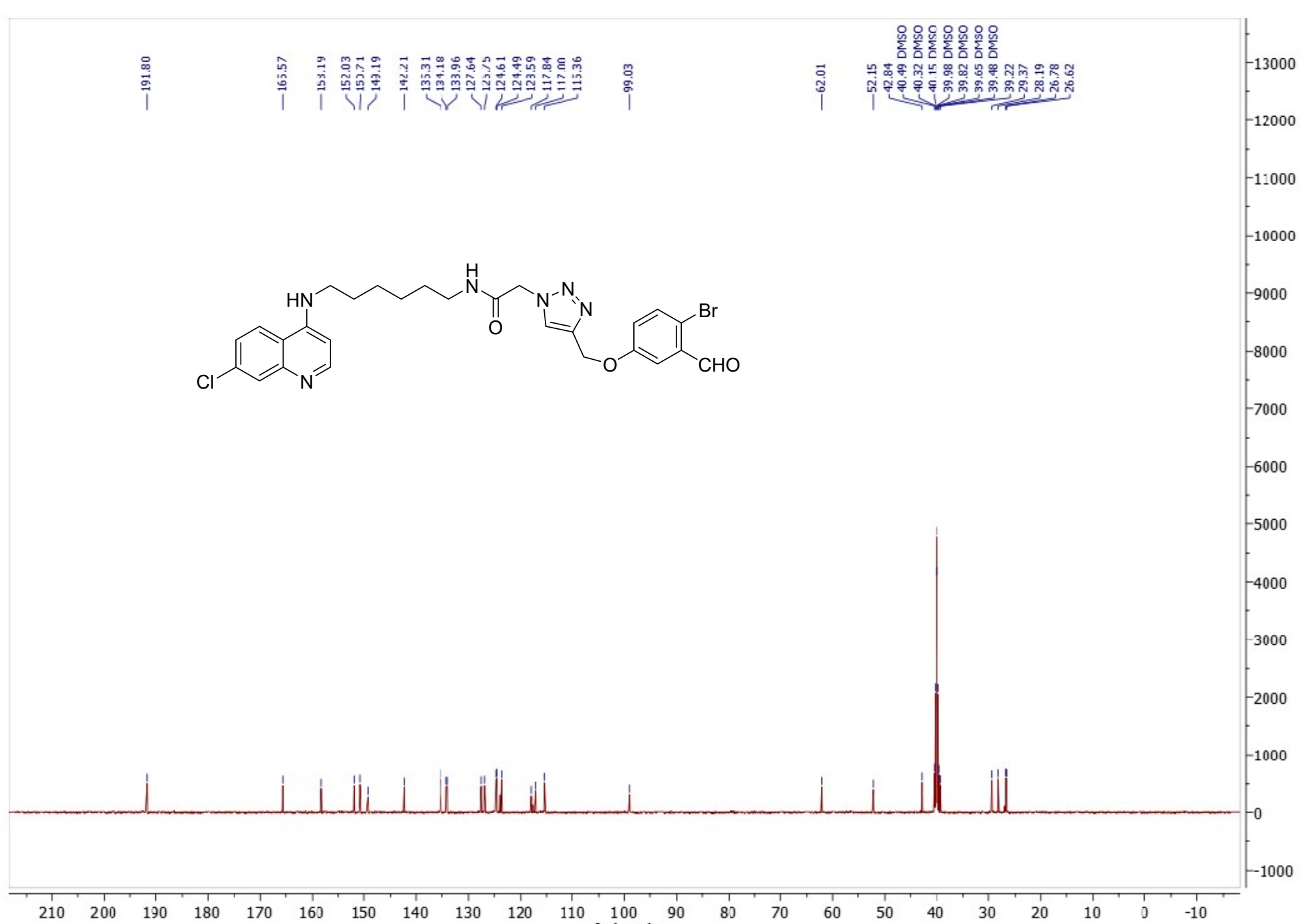

^{1}H NMR of Compound 5b:

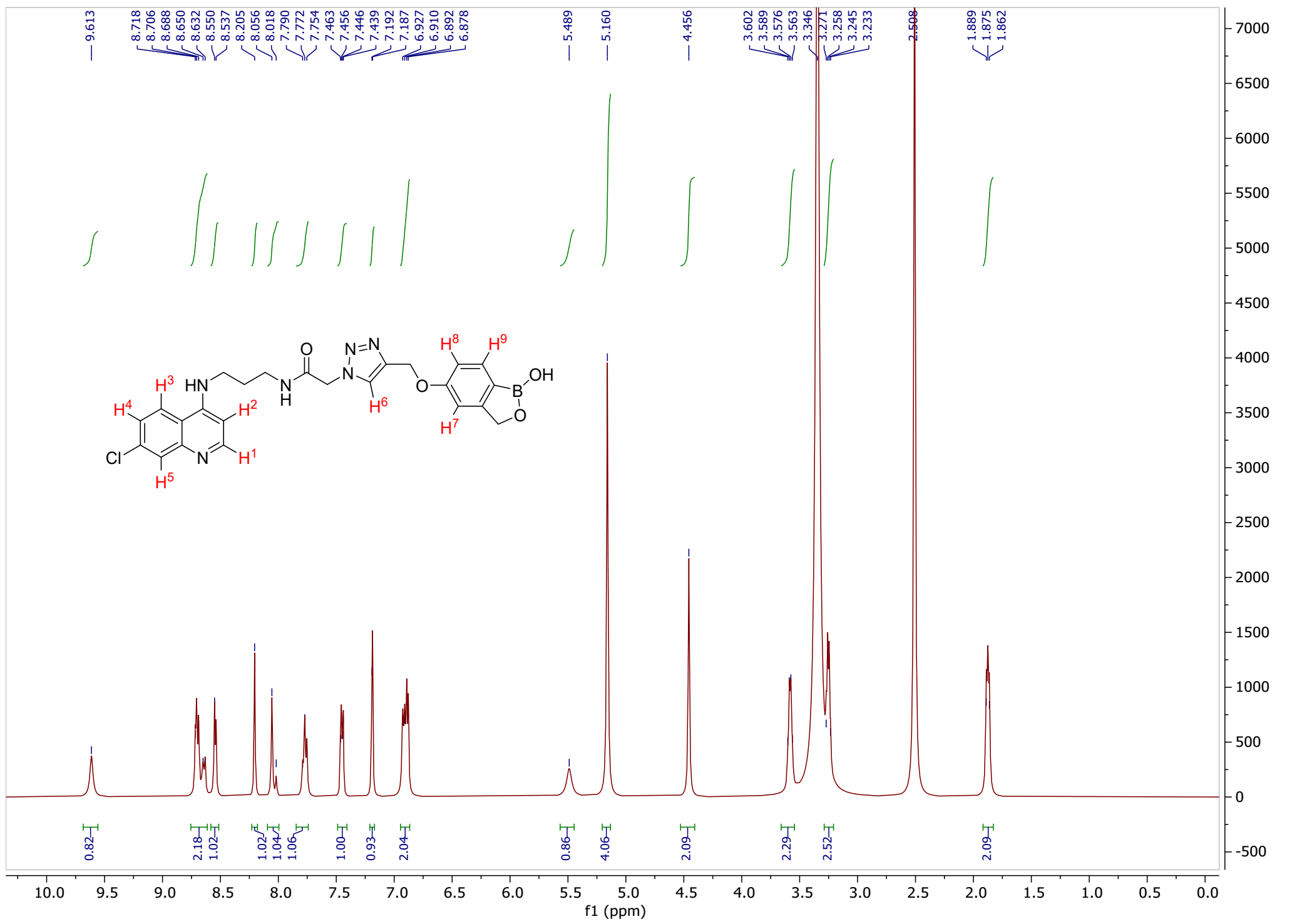

^{13}C NMR of Compound 5b:

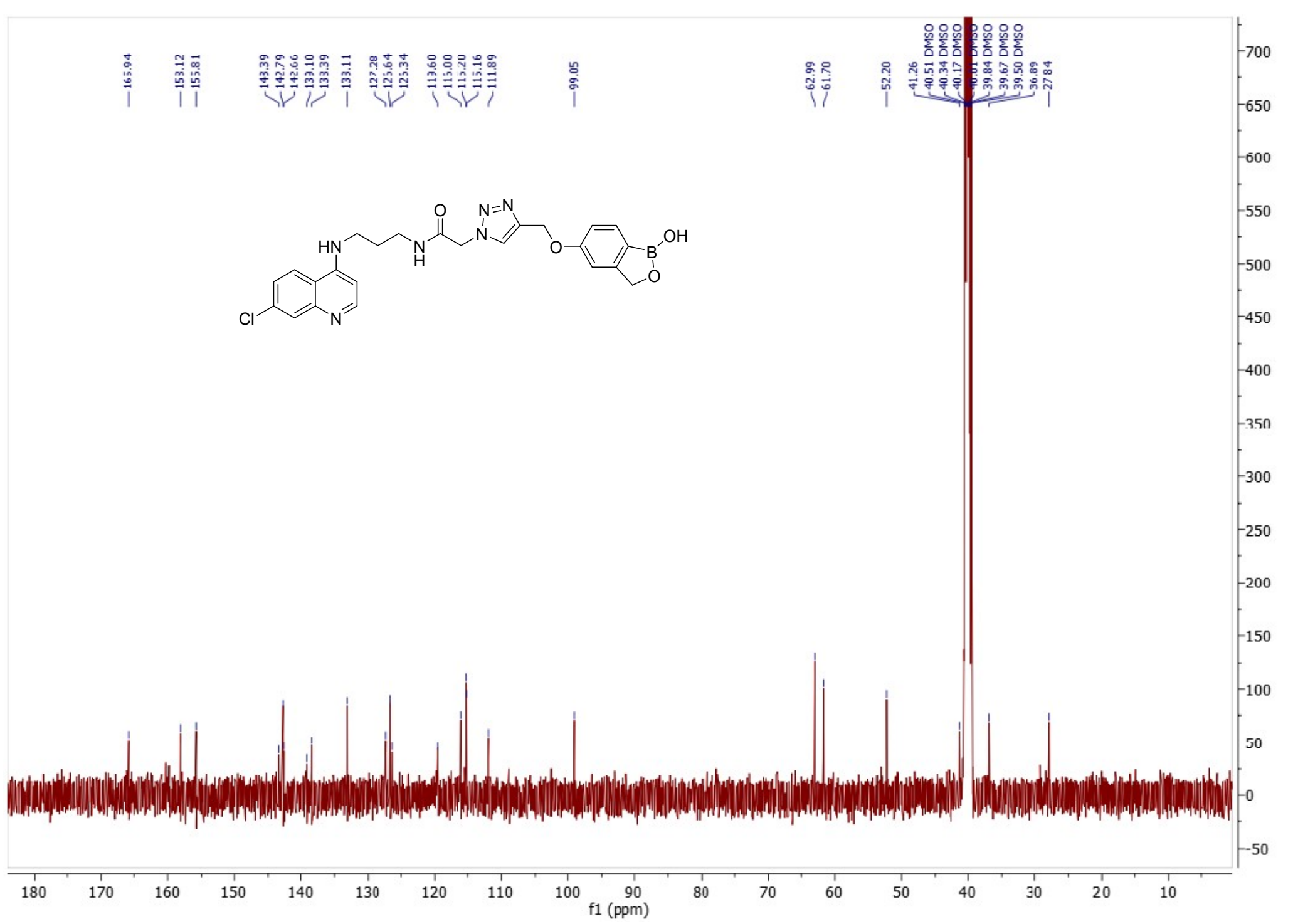

^{1}H NMR of Compound 5d:

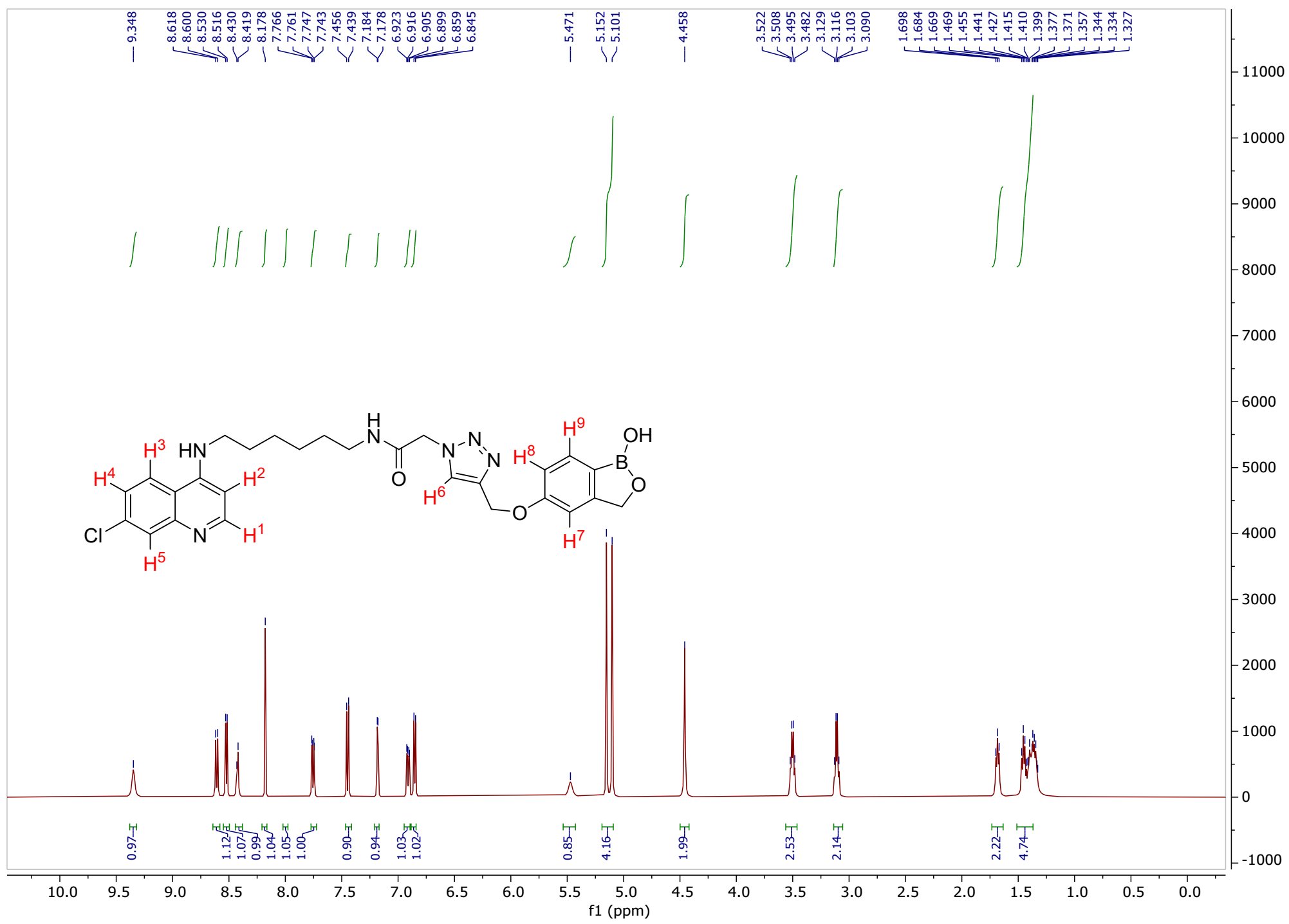

^{13}C NMR of Compound 5d:

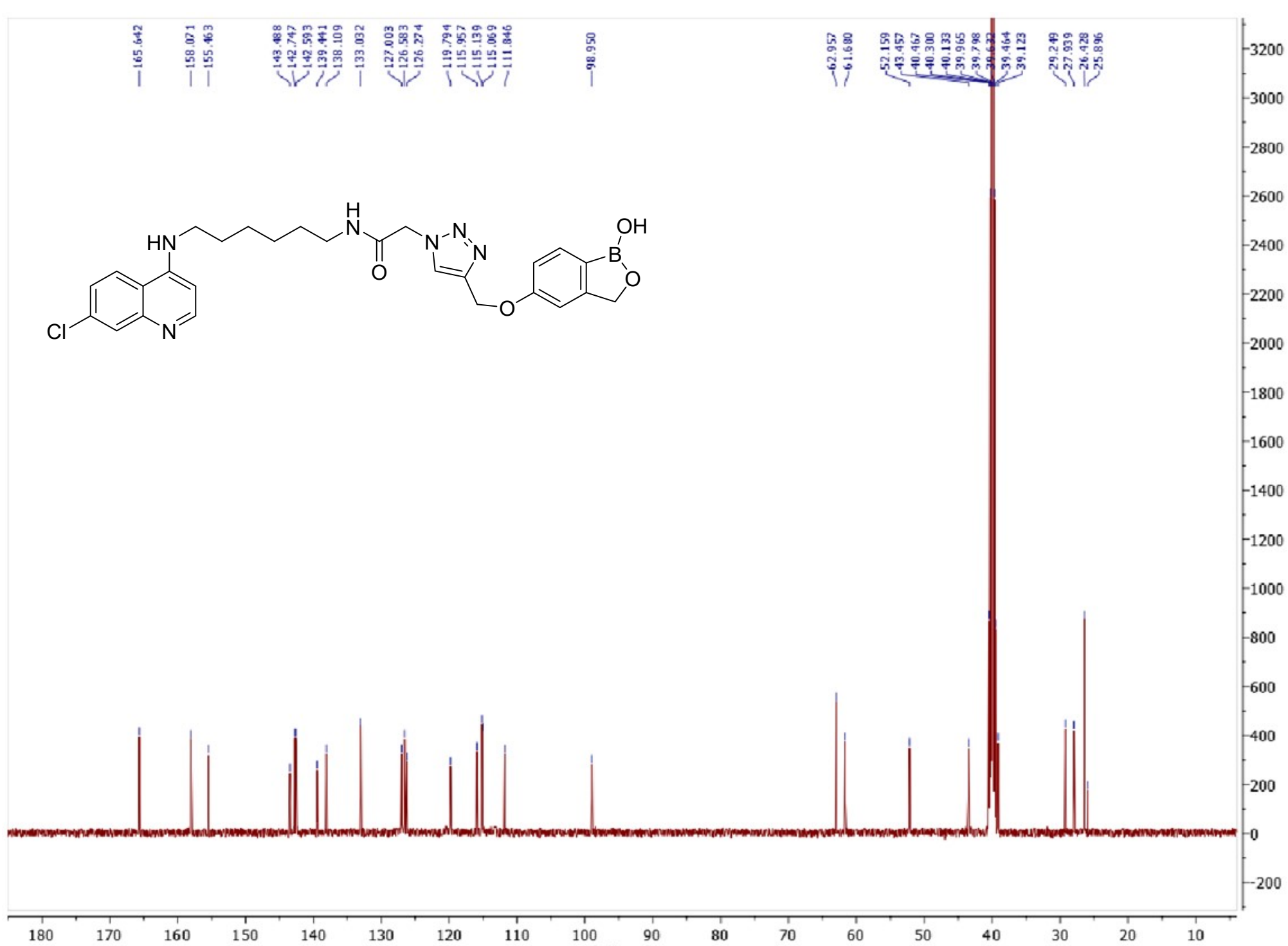

^{1}H NMR of Compound 6a:

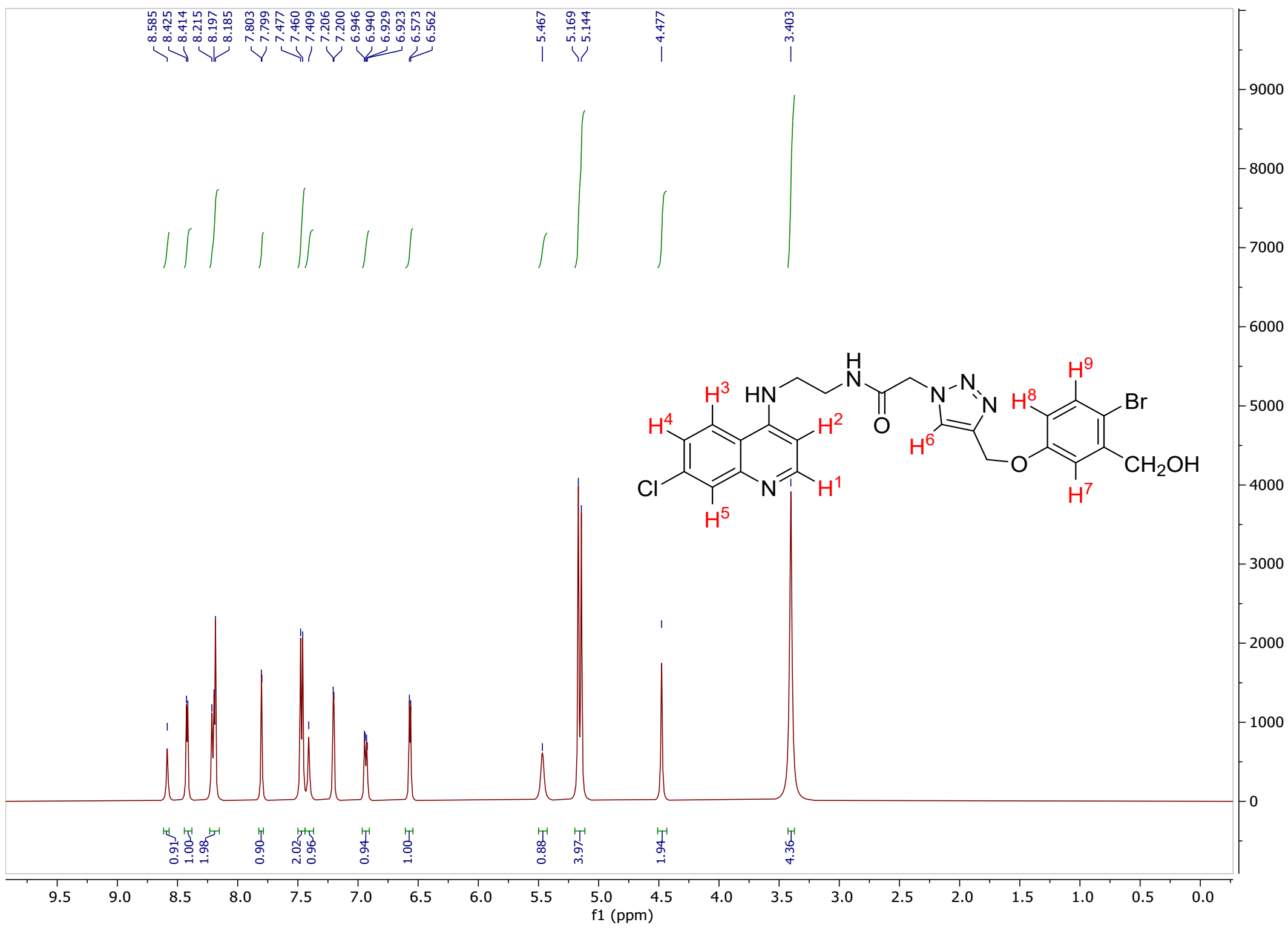

^{13}C NMR of Compound 6a:

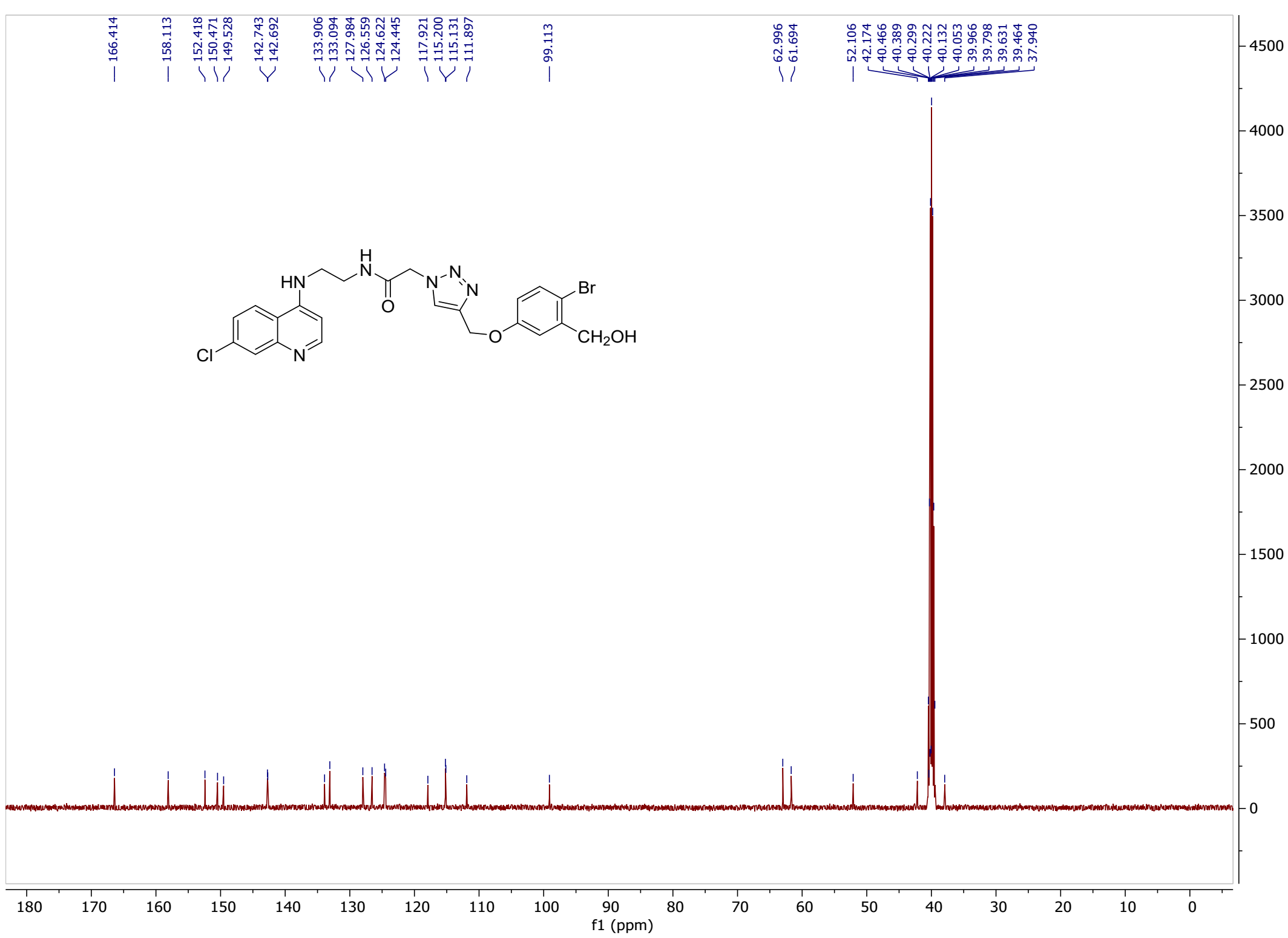

^{1}H NMR of Compound 6c:

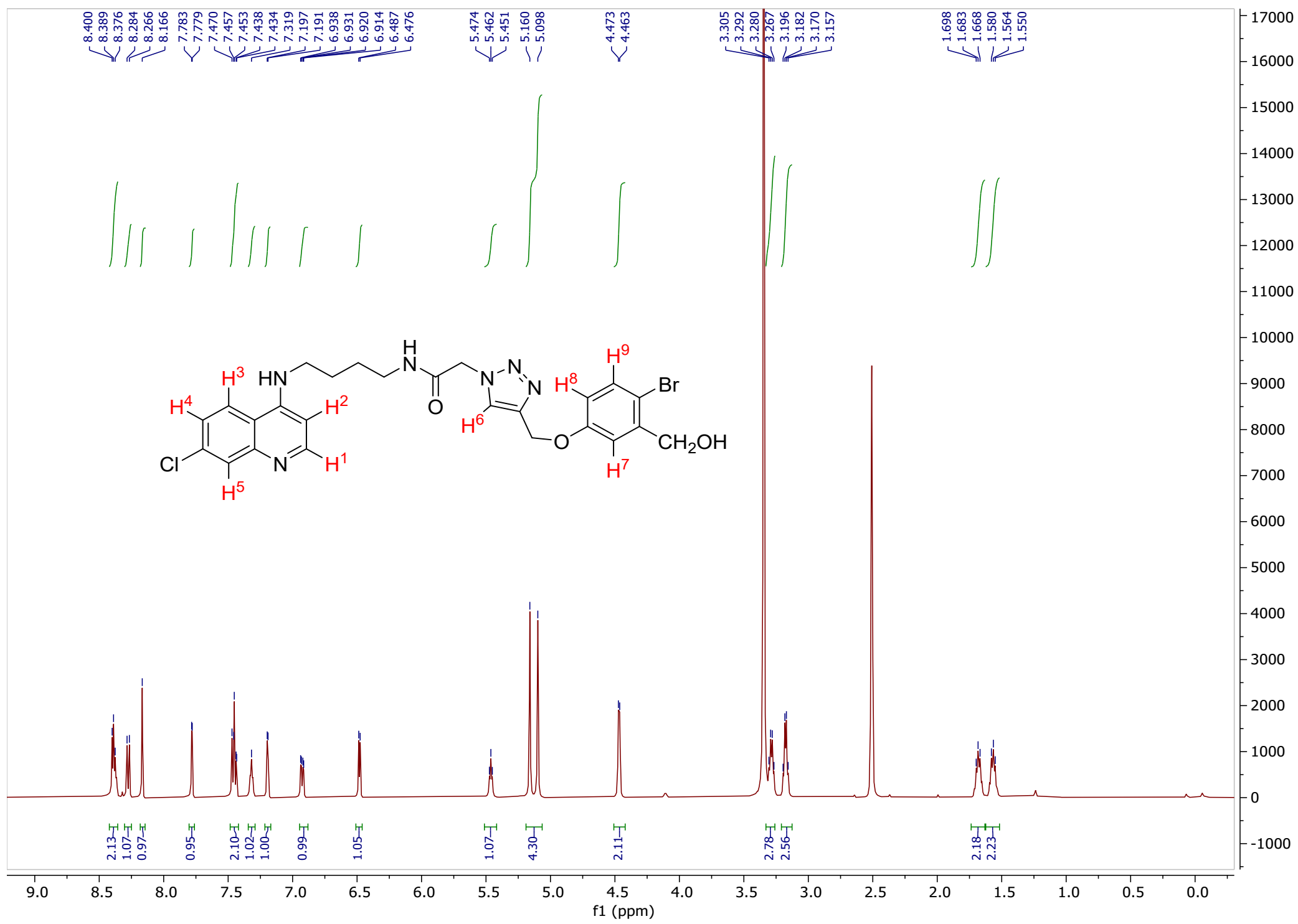

^{13}C NMR of Compound 6c:

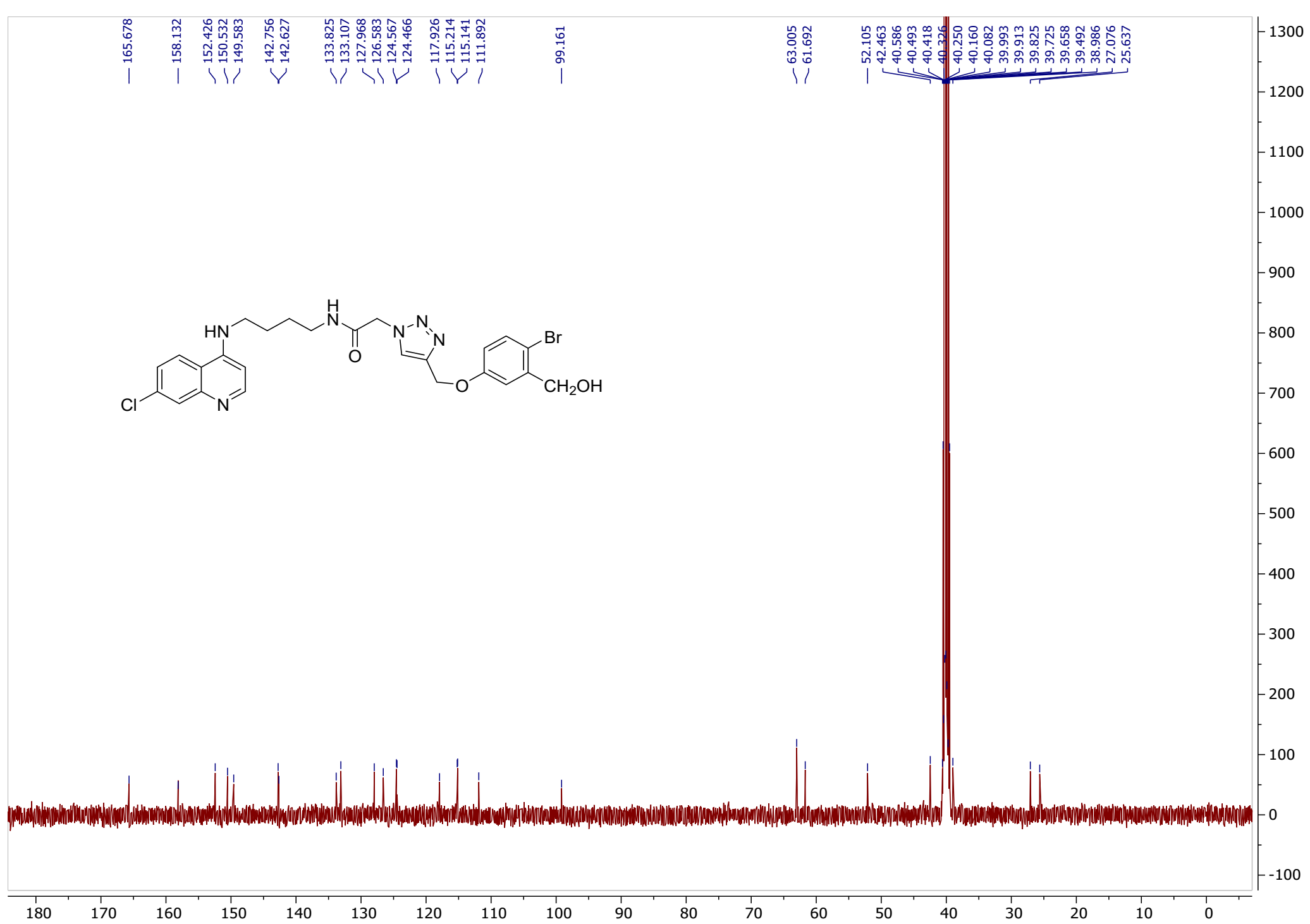

Computational Methods

Target Selection

Plasmodium falciparum cleavage and polyadenylation specificity factor subunit 3 (PfCPSF3) has been reported as a validated antimalarial target for benzoxaborole containing antimalarial compounds. Thus, PfCPSF3 was selected as the potential target for the synthesized benzoxaborole–4-aminoquinoline hybrids for the computational studies [1,2].

Homology modelling, structure validation and analysis of PfCPSF3.

An experimentally determined crystal structure of PfCPSF3 has not yet been deposited in the RCSB Protein Data Bank (RCSB PDB) [3], thus its three-dimensional (3D) structure was modelled. The FASTA sequence of PfCPSF3, isolate 3D7, was retrieved from UniProtKB under accession number Q8IL83. The query sequence was used to perform a homology search against the NCBI BLAST database to identify suitable structural templates. The crystal structure of *Cryptosporidium hominis* CPSF3 (PDB ID: 6Q55) with a zinc coordinated catalytic site was selected as the modelling template due to its high sequence identity with the query sequence (~51% identity). Sequence alignment between the PfCPSF3 query sequence and the template structure was performed using the Clustal Omega algorithm within the BLAST framework and further refined in Maestro using the Prime module (Schrödinger Release 2023-2: Schrödinger, LLC, New York, NY, 2023).

The 3D structure of PfCPSF3 was subsequently constructed using the knowledge-based method in Prime in homology modelling module, with *C. hominis* CPSF3 serving as the structural template. The resulting model was structurally optimised using conjugate gradient energy minimisation with OPLS4 force field for up to 5000 iterations to remove steric clashes and relax the structure, with convergence reached after 2850 iterations. The minimised structure was subsequently subjected to loop refinement using the Prime module to improve the conformational accuracy of regions with insertions or low structural conservation.

Validation of modelled structure

To assess the quality, reliability and stereochemical quality of the of the modelled PfCPSF3 structure, ProSA-web server (https://prosa.services.came.sbg.ac.at/prosa.php) [4] and MolProbity sever (https://swissmodel.expasy.org/assess) [5] were employed.

Compound Preparations (LigPrep)

The molecular structures of the synthesised compounds (**8a–8e**) were constructed using ChemBioDraw Ultra 14.0 and exported in SDF format. The structures were subsequently imported into Maestro (Schrödinger, LLC) for ligand preparation. Three-dimensional geometries were generated and energy-minimised under the OPLS4 force field using the LigPrep module with default parameters. Ionisation states were assigned using Epik at a target pH of 7.0 ± 2.0. The prepared ligands were subsequently used for induced-fit molecular docking.

Protein Preparations

The validated modelled structure of PfCPSF3 was prepared using the Protein Preparation Wizard workflow in Maestro (Schrödinger, LLC). The preparation procedure included the addition of hydrogen atoms, assignment of bond orders, and calculation of partial atomic charges using the OPLS4, followed by protonation state assignment. The prepared structure was subsequently subjected to restrained energy minimisation in vacuum using the OPLS4 force field [6]. Minimisation was terminated when the root-mean-square deviation (RMSD) of atomic positions reached a maximum cut off of 0.30 Å. During this process, hydrogen atoms were freely minimised while heavy atoms were restrained, allowing relaxation of strained bonds, angles, and steric clashes.

Receptor Grid Generation

The active site of PfCPSF3 was defined around the centroid of binding site residues Asn463, His446, His468, His203, His65, His63, His68, Asp224, Asp67, Phe286, Met382, Thr409, Asn159, Ser444, Val410, Arg290, Thr406, Tyr408, Asp470/Asn470, Tyr252, His36/Tyr36, Gly381, Val285, Thr251, Arg17, Val15, Gly34, Ile35, Pro37, Ala38, and Leu288.

Induced Fit Molecular Docking

To incorporate the structural flexibility of both the receptor and ligands during the binding process, induced-fit docking (IFD) implemented in the Schrödinger Suite was employed [7]. The docking grid was defined by positioning it at the centroid of residues comprising the active site. The IFD procedure was performed through a sequential multi-step workflow. In the first stage, ligands were docked into the receptor binding pocket using the Glide standard precision (SP) algorithm to generate multiple potential binding conformations. To enhance conformational sampling, the van der Waals radii of ligand and receptor atoms were scaled by 0.50, while the Coulomb–van der Waals interaction cutoff was adjusted to permit softer potential interactions.

For each predicted ligand pose, amino acid side chains located within 5.0 Å of the ligand were refined and subjected to energy minimisation using the Prime module, allowing structural adjustments of the binding pocket to accommodate ligand-induced conformational changes. The top 20 poses generated from this step were subsequently re-docked into their corresponding refined receptor structures using the Glide extra precision (XP) mode. The resulting complexes were evaluated using the IFD score, which integrates contributions from GlideScore and Prime energy terms. For each ligand–protein complex, the binding pose associated with the lowest IFD score was selected as the final predicted conformation.

Molecular Dynamics Simulation

Molecular dynamics (MD) simulation helps to determine the stability of a ligand–protein complex and examine the interactions between the protein and ligand over time. MD simulations of compounds **8a–8e** in complex with PfCPSF3 wild-type and mutants obtained from the IFD were performed using the Desmond module implemented in Schrödinger Maestro 2023-2. System preparation was carried out using the System Builder tool integrated within Desmond. The protein–ligand complexes were embedded in an orthorhombic simulation box with a dimension of 10 Å and solvated using the SPC water model [8]. The systems were neutralized by the addition of Cl^- counter ions. Energy minimization was performed prior to the production run with the OPLS4 force field [6]. MD simulations parameters were set using the Molecular Dynamics panel in Desmond. A simulation time of 100 ns was performed, and trajectories were recorded at 100 ps intervals, yielding approximately 1000 frames. The systems were equilibrated using the default Desmond relaxation protocol. Production simulations were carried out under the NPT ensemble at 300 K and 1.01325 bar, using the Nosé–Hoover chain thermostat and the Martyna–Tobias–Klein barostat [9,10]. The Simulations Interactions Diagram (SID) panel was used to analyse trajectories, including parameters such as RMSD and protein–ligand contacts, while 3D binding mode interactions were generated using Schrödinger Maestro.

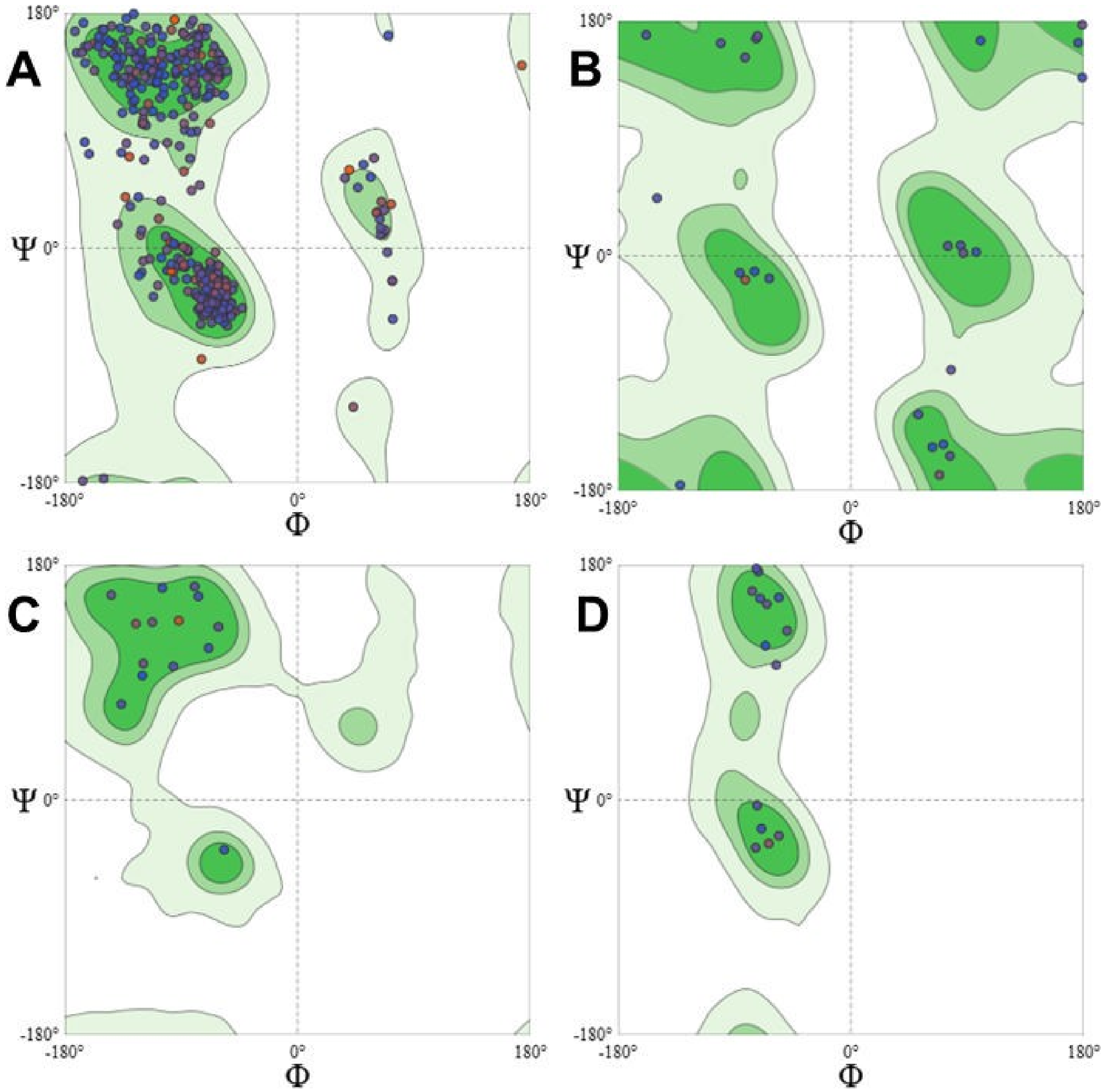

Figure S1: Ramachandran plot analysis of the modeled PfCPSF3 structure after loop refinement and energy minimization. Plots show (a) all residues, (b) glycine, (c) pre-proline and (d) proline. MolProbity Score of 1.31 (acceptable distribution Z-score < 2) and a QMEANDisCo Global score of 0.77±0.05.

Table S1. Ramachandran plot statistics for the modeled PfCPSF3 structure after loop refinement and energy minimization.

Region of Ramachandran plot	Residues (%)
Percentage of residues in favoured regions	93.65
Percentage of residues in allowed regions (favoured + additionally allowed)	99.12
Outlier regions	0.88
Clash score	0.27

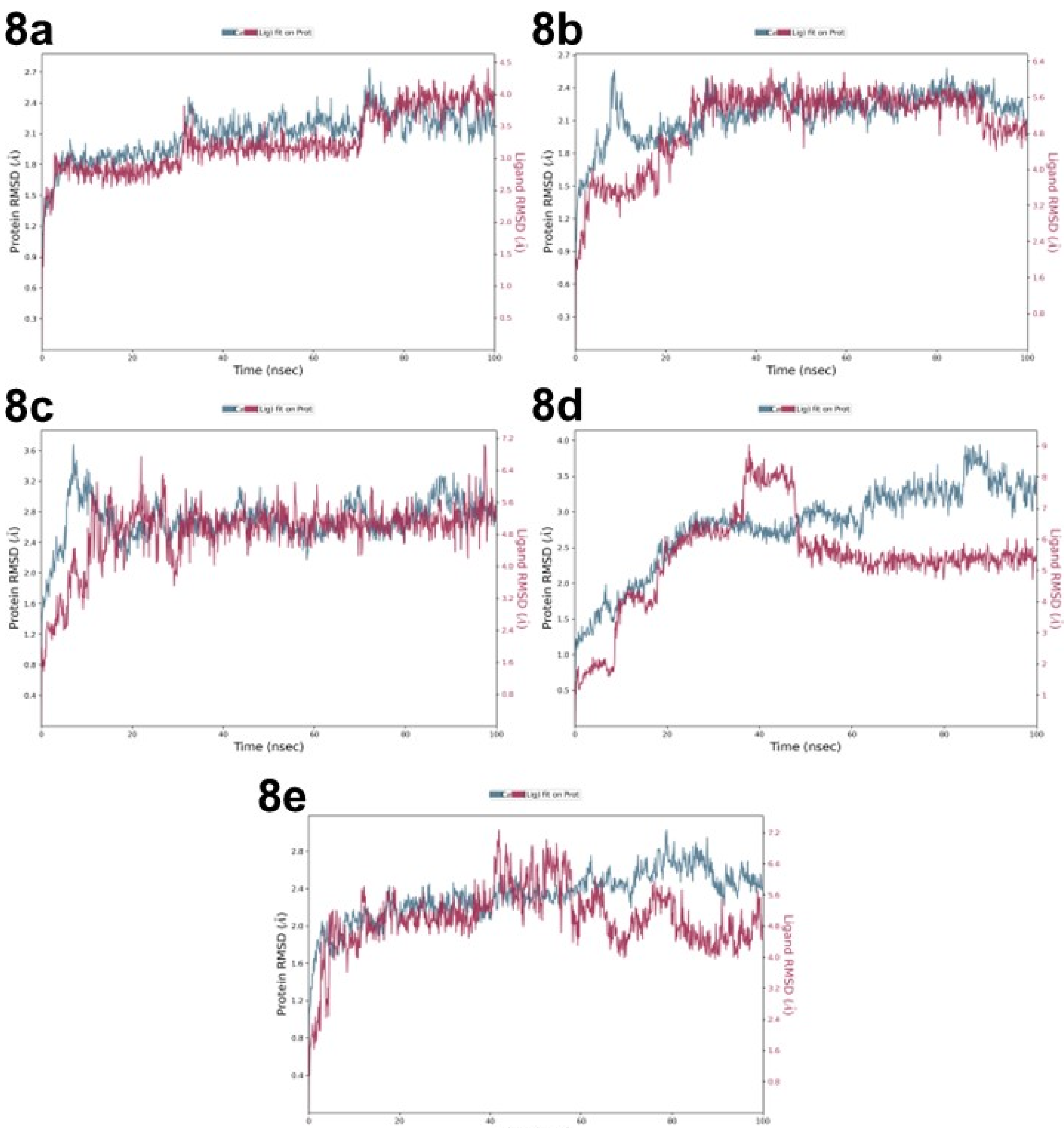

Figure S2: Root mean square deviations (RSMD) of Cα atoms of PfCPSF3-WT complexes over the 100ns MD simulations.

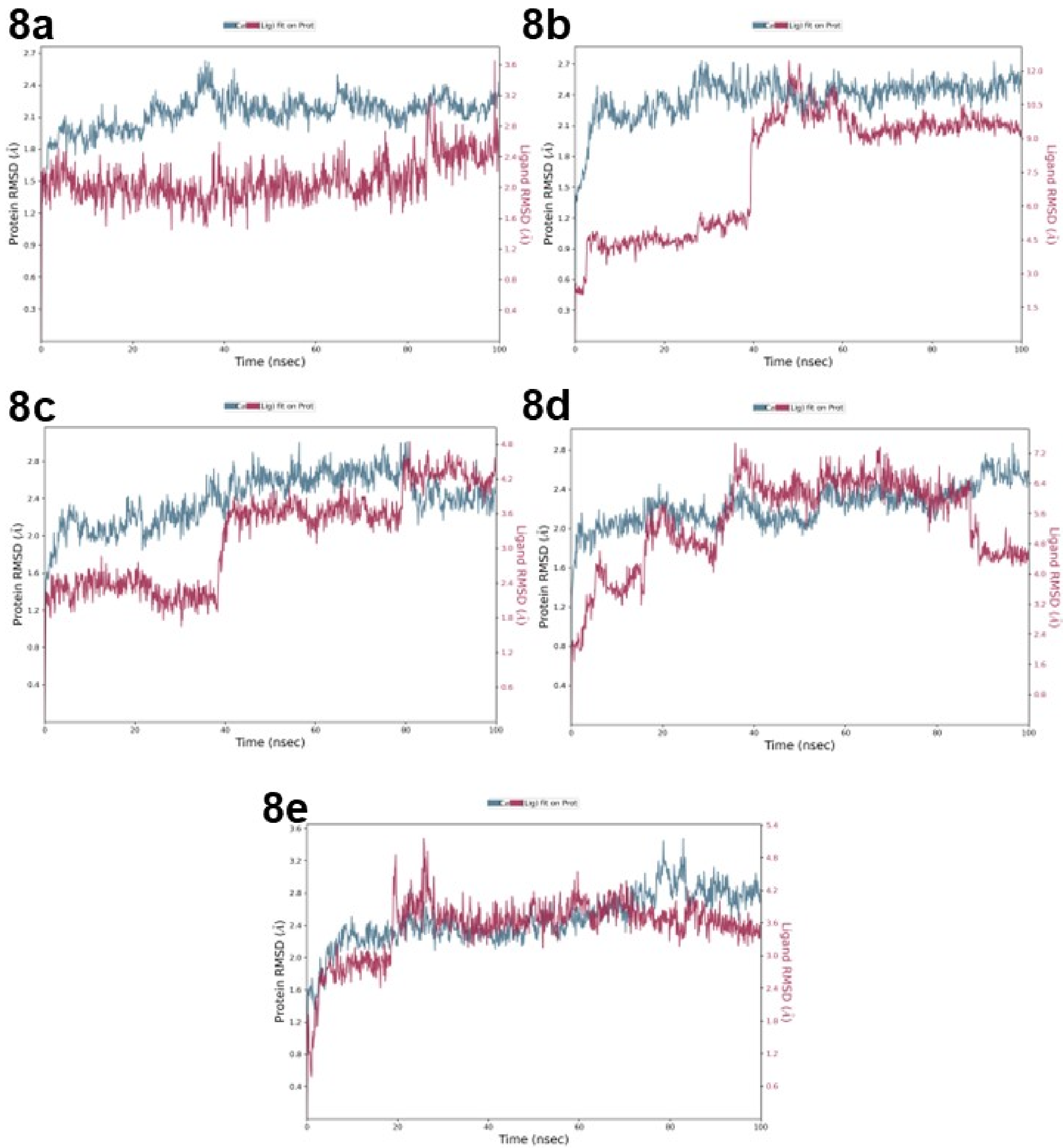

Figure S3: Root mean square deviations (RSMD) of Cα atoms of PfCPSF3-MT complexes over the 100ns MD simulations.

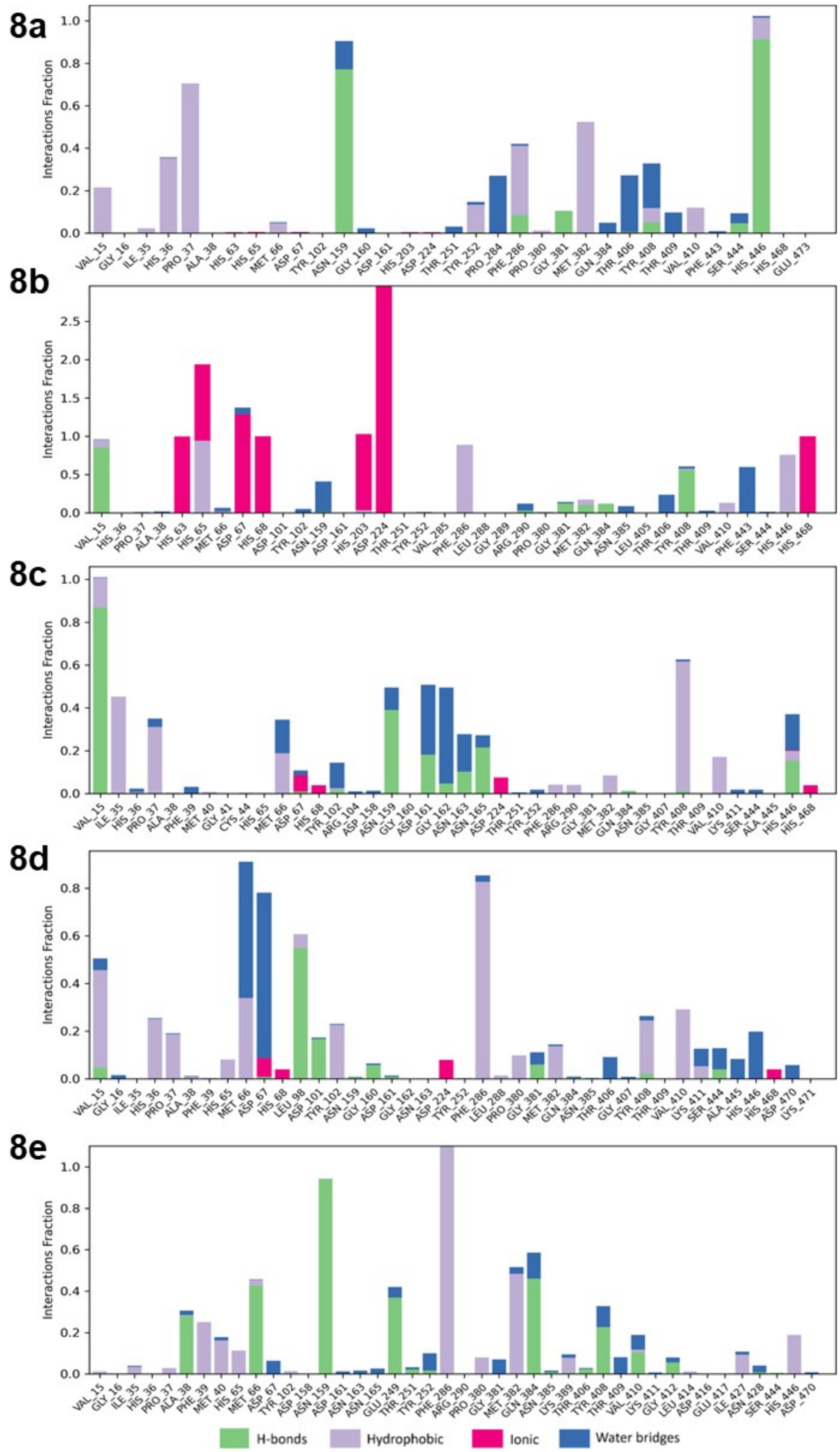
8a
8b
8c
8d
8e
Interactions Fraction
H-bonds
Hydrophobic
Ionic
Water bridges

Figure S4. Fraction summary of protein-ligand contacts for PfCPSF3-WT normalized over the 100ns simulation time. Interaction fractions exceeding 1.0 indicate that a residue forms multiple contacts with the ligand.

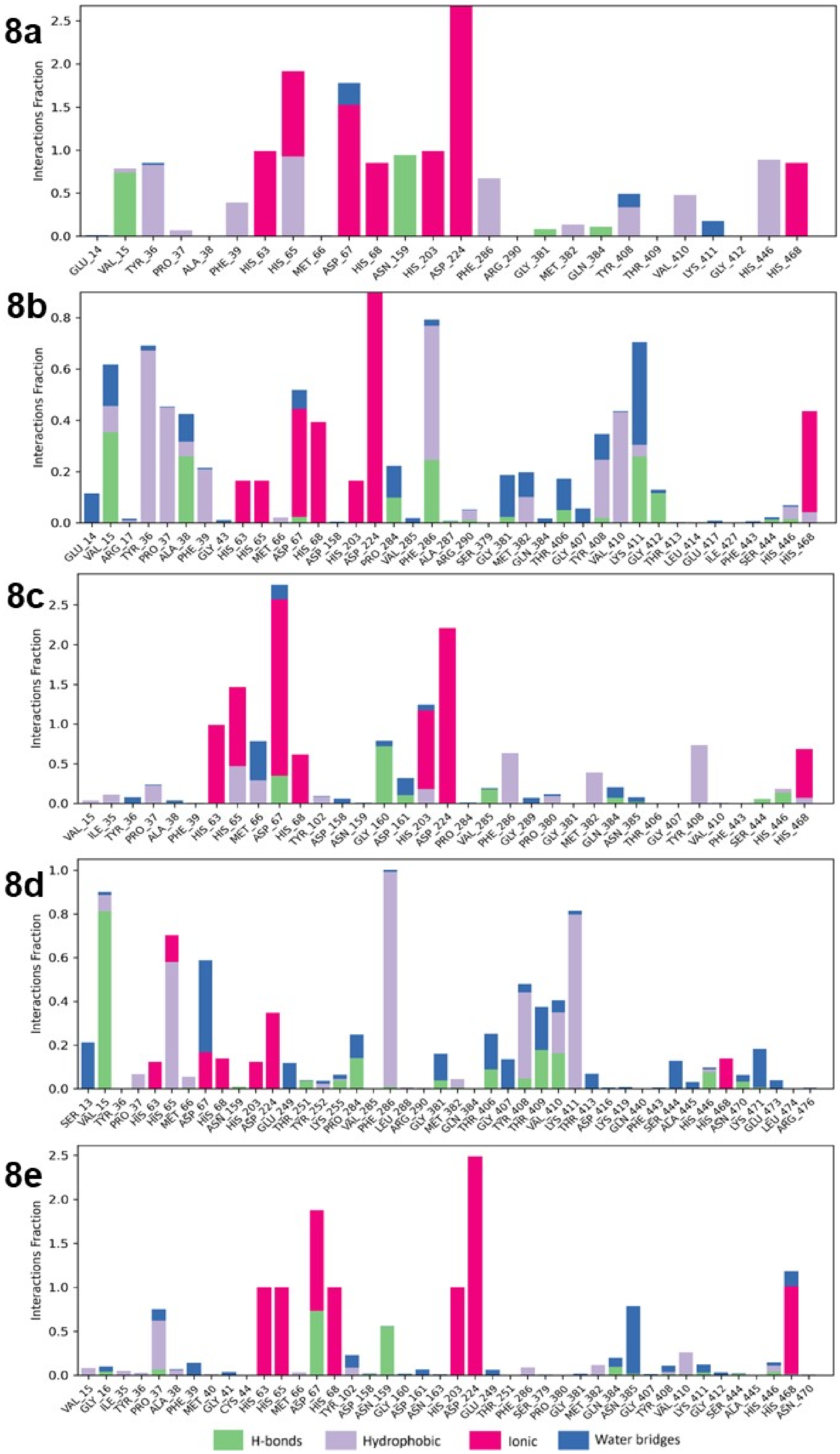

8a
8b
8c
8d
8e
Interactions Fraction
H-bonds
Hydrophobic
Ionic
Water bridges

Figure S5. Fraction summary of protein-ligand contacts for PfCPSF3-MT normalized over the 100ns simulation time. Interaction fractions exceeding 1.0 indicate that a residue forms multiple contacts with the ligand.

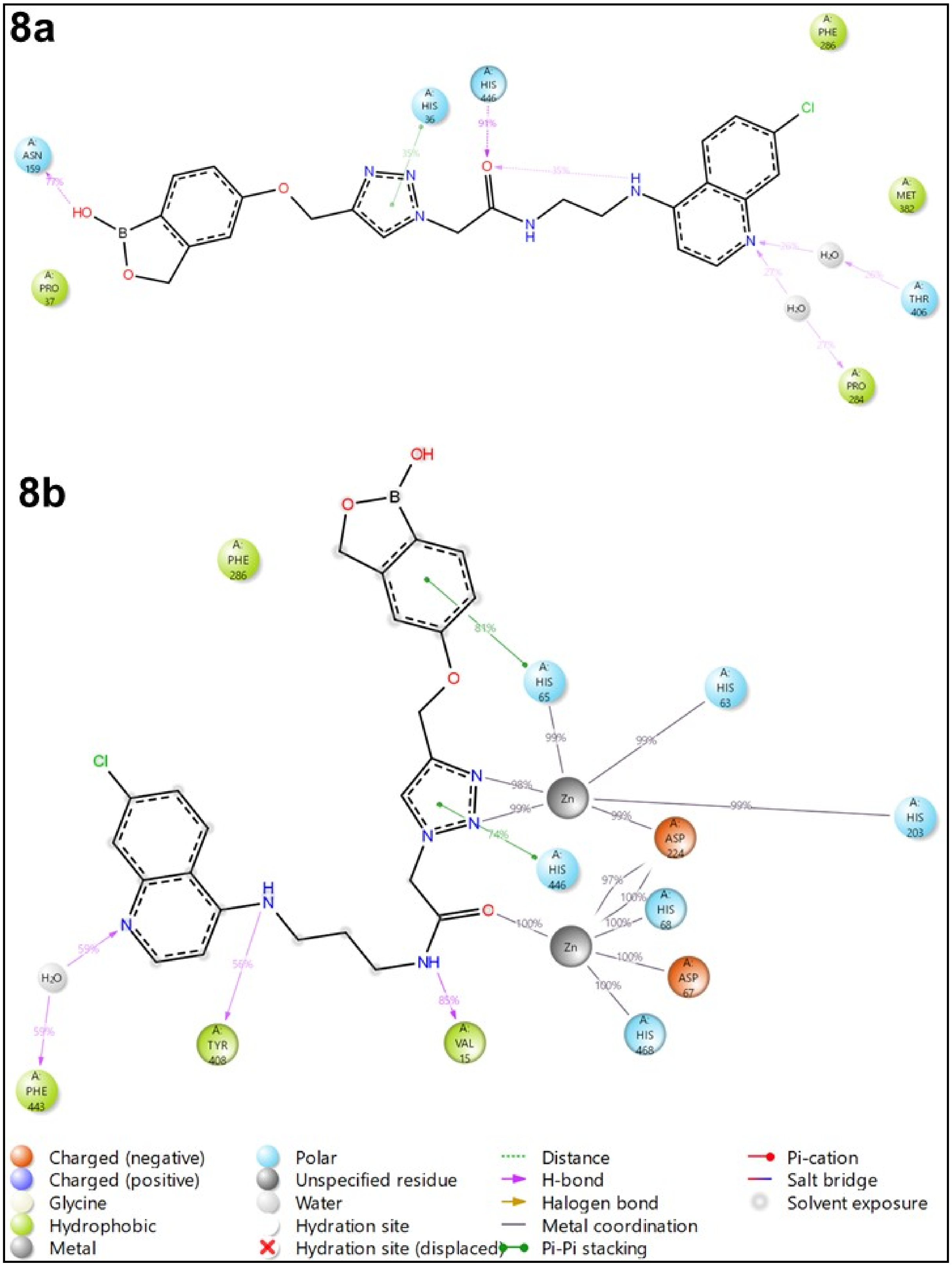
8a
A: PHE 286
A: HIS 446
A: HIS 36
A: ASN 159
91%
35%
35%
77%
Cl
O
N
N
N
H
N
HO
B
O
N
H
N
A: MET 382
A: PRO 37
26%
H2O
26%
27%
H2O
A: THR 406
27%
A: PRO 284
8b
OH
B
O
A: PHE 286
81%
O
A: HIS 65
A: HIS 63
Cl
99%
99%
N
98%
Zn
99%
N
99%
99%
A: HIS 203
N
74%
A: ASP 224
A: HIS 446
97%
H
N
100%
A: HIS 68
N
O
100%
100%
Zn
59%
56%
100%
H2O
NH
A: ASP 67
100%
59%
85%
A: TYR 408
A: VAL 15
A: HIS 468
A: PHE 443
Charged (negative)
Charged (positive)
Glycine
Hydrophobic
Metal
Polar
Unspecified residue
Water
Hydration site
Hydration site (displaced)
Distance
H-bond
Halogen bond
Metal coordination
Pi-Pi stacking
Pi-cation
Salt bridge
Solvent exposure

Figure S6: 2D protein–ligand interaction maps of PfCPSF3-WT complexes with compounds 8a, and 8b, highlighting interactions observed in >20% of the 100 ns simulation.

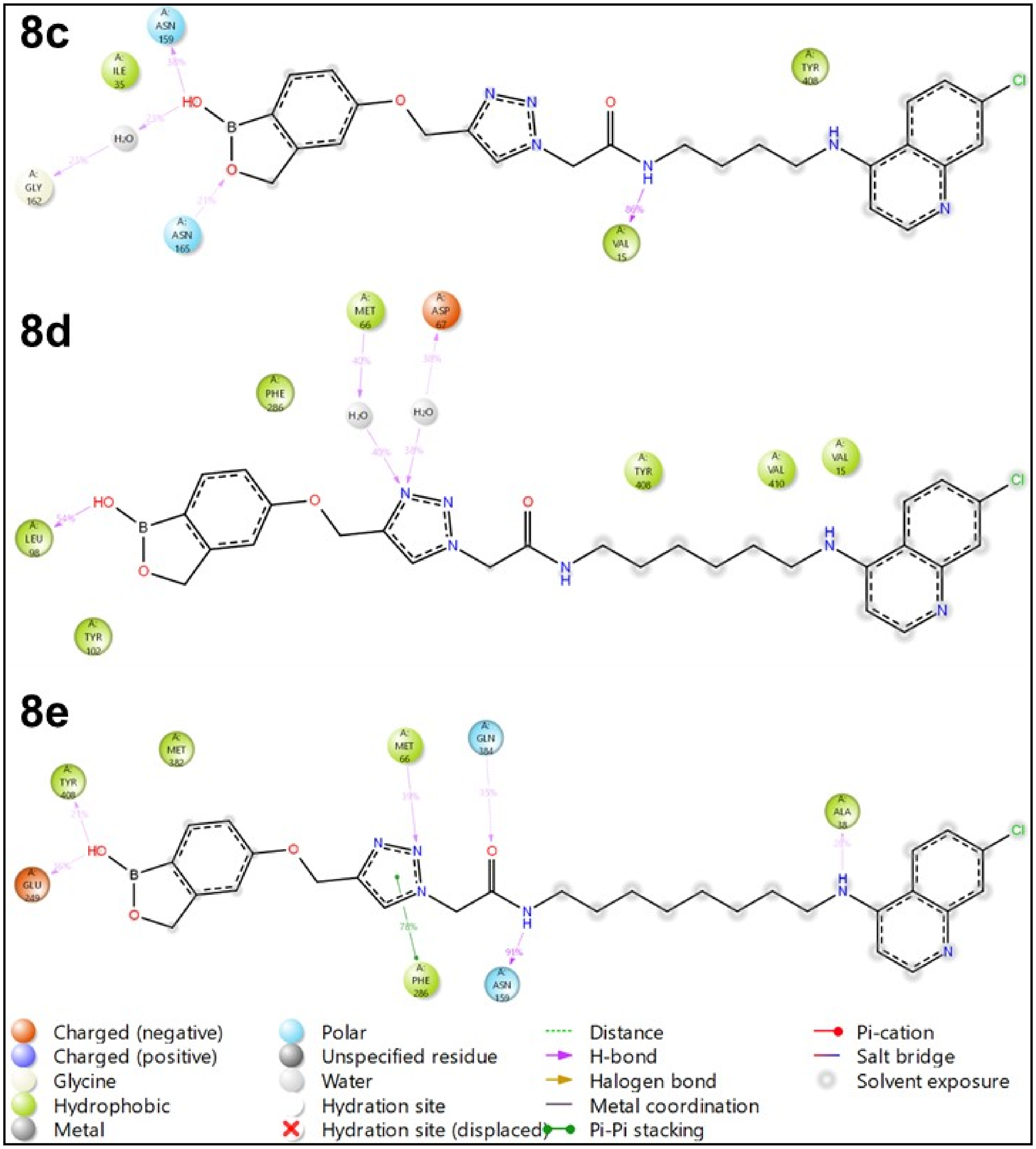

Figure S6-continued: 2D protein–ligand interaction maps of PfCPSF3-WT complexes with compounds 8c, 8d and 8e highlighting interactions observed in >20% of the 100 ns simulation.

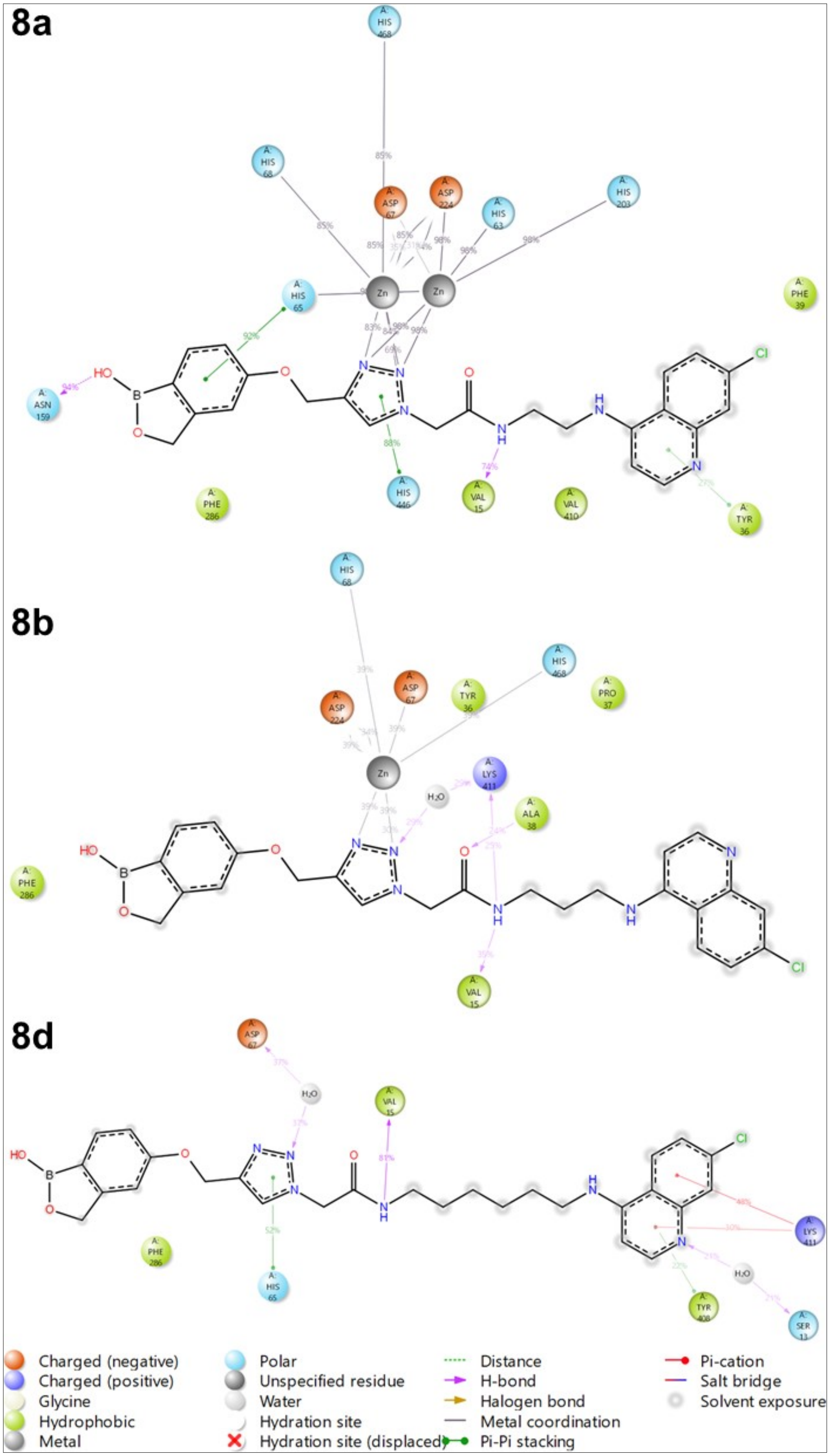
8a
8b
8d
Charged (negative)
Charged (positive)
Glycine
Hydrophobic
Metal
Polar
Unspecified residue
Water
Hydration site
Hydration site (displaced)
Distance
H-bond
Halogen bond
Metal coordination
Pi-Pi stacking
Pi-cation
Salt bridge
Solvent exposure

Figure S7: 2D protein–ligand interaction maps of PfCPSF3-MT complexes with compounds 8a, 8b, and 8d, highlighting interactions observed in >20% of the 100 ns simulation.

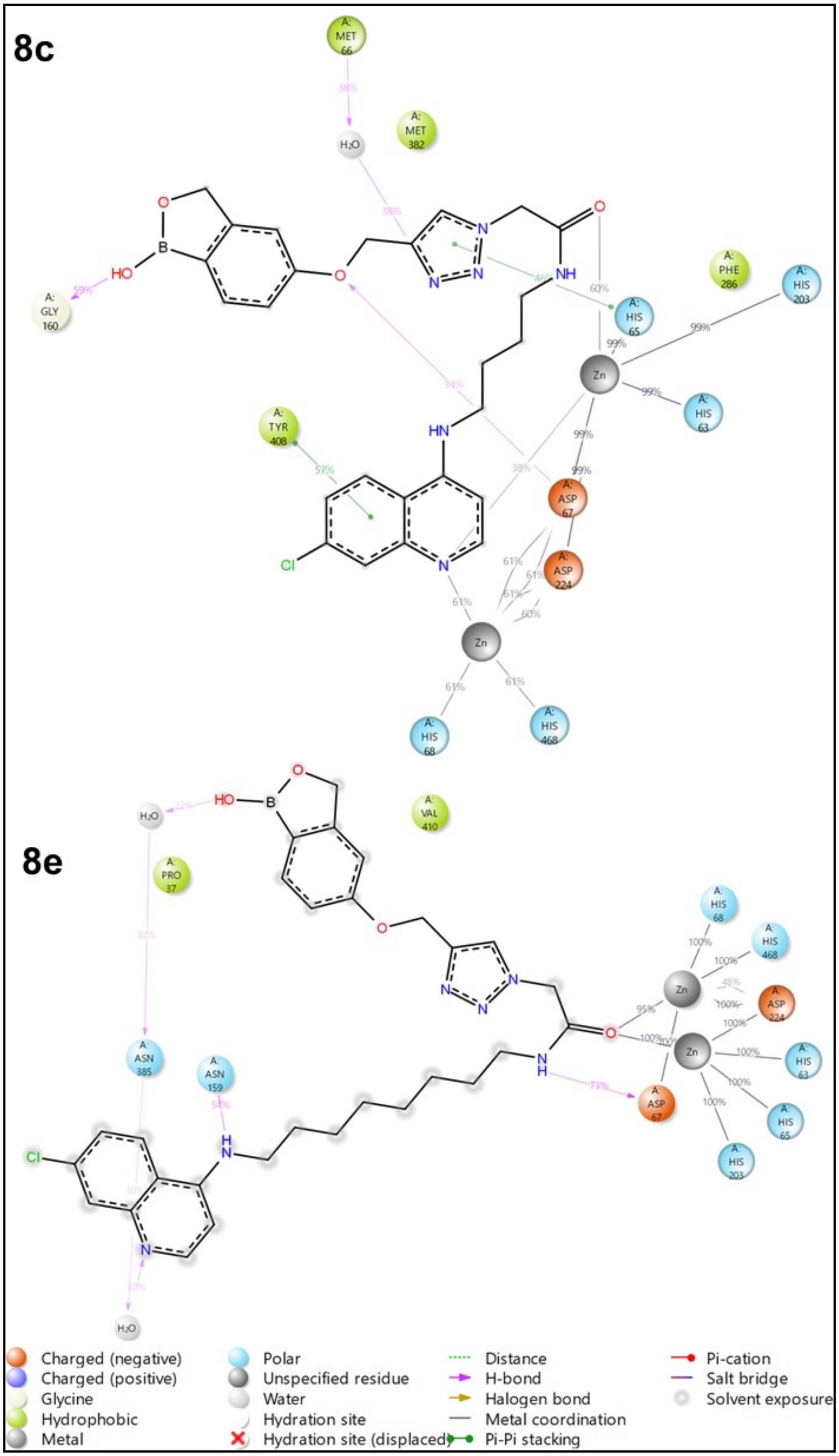
8c
8e
Charged (negative)
Charged (positive)
Glycine
Hydrophobic
Metal
Polar
Unspecified residue
Water
Hydration site
Hydration site (displaced)
Distance
H-bond
Halogen bond
Metal coordination
Pi-Pi stacking
Pi-cation
Salt bridge
Solvent exposure

Figure S7-continued: 2D protein–ligand interaction maps of PfCPSF3-MT complexes with compounds 8c and 8e highlighting interactions observed in >20% of the 100 ns simulation.

References

[1] E. Sonoiki, C.L. Ng, M.C.S. Lee, D. Guo, Y.-K. Zhang, Y. Zhou, M.R.K. Alley, V. Ahyong, L.M. Sanz, M.J. Lafuente-Monasterio, A potent antimalarial benzoxaborole targets a Plasmodium falciparum cleavage and polyadenylation specificity factor homologue, Nat. Commun. 8 (2017) 14574.

[2] S.K.M. V., W. Wesley, L. Jenny, Z. Yong-Kang, E.E. E., C.R. A., P.J. J., F.Y. R., D.J. L., R.P. J., Plasmodium falciparum Resistance to a Lead Benzoxaborole Due to Blocked Compound Activation and Altered Ubiquitination or Sumoylation, MBio 11 (2020) 10.1128/mbio.02640-19. https://doi.org/10.1128/mbio.02640-19.

[3] S.K. Burley, D.W. Piehl, B. Vallat, C. Zardecki, RCSB Protein Data Bank: supporting research and education worldwide through explorations of experimentally determined and computationally predicted atomic level 3D biostructures, IUCrJ 11 (2024) 279–286.

[4] M. Wiederstein, M.J. Sippl, ProSA-web: interactive web service for the recognition of errors in three-dimensional structures of proteins, Nucleic Acids Res. 35 (2007) W407–W410.

[5] A.M. Waterhouse, G. Studer, X. Robin, S. Bienert, G. Tauriello, T. Schwede, The structure assessment web server: for proteins, complexes and more, Nucleic Acids Res. 52 (2024) W318–W323.

[6] C. Lu, C. Wu, D. Ghoreishi, W. Chen, L. Wang, W. Damm, G.A. Ross, M.K. Dahlgren, E. Russell, C.D. Von Bargen, OPLS4: improving force field accuracy on challenging regimes of chemical space, J. Chem. Theory Comput. 17 (2021) 4291–4300.

[7] W. Sherman, T. Day, M.P. Jacobson, R.A. Friesner, R. Farid, Novel procedure for modeling ligand/receptor induced fit effects, J. Med. Chem. 49 (2006) 534–553.

[8] P. Mark, L. Nilsson, Structure and dynamics of the TIP3P, SPC, and SPC/E water models at 298 K, J. Phys. Chem. A 105 (2001) 9954–9960.

[9] S. Nosé, A unified formulation of the constant temperature molecular dynamics methods, J. Chem. Phys. 81 (1984) 511–519.

[10] G.J. Martyna, M.E. Tuckerman, D.J. Tobias, M.L. Klein, Explicit reversible integrators for extended systems dynamics, Mol. Phys. 87 (1996) 1117–1157.
